# Identification of a Novel CD8 T Cell Epitope Derived from *Plasmodium berghei* Protective Liver-Stage Antigen

**DOI:** 10.3389/fimmu.2018.00091

**Published:** 2018-01-29

**Authors:** Alexander Pichugin, Stasya Zarling, Leah Perazzo, Patrick Emmet Duffy, Hidde Lolke Ploegh, Urszula Krzych

**Affiliations:** ^1^Department of Cellular Immunology, Malaria Vaccine Branch, Walter Reed Army Institute of Research, Silver Spring, MD, United States; ^2^Laboratory of Malaria Immunology and Vaccinology, National Institute of Allergy and Infectious Diseases, (NIH), Rockville, MD, United States; ^3^Program in Cellular and Molecular Medicine, Division of Molecular Biology, Department of Medicine, Boston Children’s Hospital, Boston, MD, United States

**Keywords:** CD8 T cells, epitope prediction, *Plasmodium*, caged MHC-tetramers, minigene

## Abstract

We recently identified novel *Plasmodium berghei* (Pb) liver stage (LS) genes that as DNA vaccines significantly reduce Pb LS parasite burden (LPB) in C57Bl/6 (B6) mice through a mechanism mediated, in part, by CD8 T cells. In this study, we sought to determine fine antigen (Ag) specificities of CD8 T cells that target LS malaria parasites. Guided by algorithms for predicting MHC class I-restricted epitopes, we ranked sequences of 32 Pb LS Ags and selected ~400 peptides restricted by mouse H-2K^b^ and H-2D^b^ alleles for analysis in the high-throughput method of caged MHC class I-tetramer technology. We identified a 9-mer H-2K^b^ restricted CD8 T cell epitope, Kb-17, which specifically recognized and activated CD8 T cell responses in B6 mice immunized with Pb radiation-attenuated sporozoites (RAS) and challenged with infectious sporozoites (spz). The Kb-17 peptide is derived from the recently described novel protective Pb LS Ag, PBANKA_1031000 (MIF4G-like protein). Notably, immunization with the Kb-17 epitope delivered in the form of a minigene in the adenovirus serotype 5 vector reduced LPB in mice infected with spz. On the basis of our results, Kb-17 peptide was available for CD8 T cell activation and recall following immunization with Pb RAS and challenge with infectious spz. The identification of a novel MHC class I-restricted epitope from the protective Pb LS Ag, MIF4G-like protein, is crucial for advancing our understanding of immune responses to Plasmodium and by extension, toward vaccine development against malaria.

## Introduction

Malaria remains one of the most serious infectious diseases that plagues residents of tropical areas such as Sub-Saharan Africa, South-East Asia, and the Indian subcontinent. Each year, more than 200 million cases of malaria, mainly caused by *Plasmodium falciparum* (Pf), are reported, with more than 400,000 deaths occurring annually (1). An effective malaria vaccine is still unavailable. The most advanced malaria vaccine, RTS,S, based on the Pf circumsporozoite protein (CSP), the major sporozoite (spz) surface antigen (Ag), induces but a modicum of protection from clinical malaria and protection is short-lived (2–4). According to the majority of results from studies of immune responses induced by RTS,S, there appears to be an absence of CSP-specific CD8 T cells (5) and that alone may limit the effectiveness of the vaccine. Therefore, addition of antigenic targets to the CSP-based vaccine, and particularly liver stage (LS) Ags that would be targeted by CD8 T cells, might rescue the modest efficacy of the otherwise well designed RTS,S vaccine.

There are many examples from animal (6–8) as well as human studies (9) that protection induced with radiation-attenuated sporozoite (RAS), the gold standard of protection, is CD8 T cell-dependent. The major sporozoite stage (SS) Ag, CSP, plays a role in RAS induced protection and results from studies with CSP-peptide TCR Tg CD8 T cells confirmed this notion (10, 11). Results from separately conducted studies have demonstrated that TRAP peptide also induces CD8 T cells that eliminate LS parasites (12). We have demonstrated that protracted protection induced by Pb RAS depends on LS Ag-specific effector and memory CD8 T cells (13). On the basis of results from an elegantly conducted study using Pb CSP-tolerized mice, it became evident that protection induced with RAS occurs in the absence of CSP-specific T cells and that it is mediated by immune responses induced by other pre-erythrocytic (PE) Ags (14). Collectively, these observations highlight the importance of many PE Ags including CSP, TRAP, and LS Ags in the generation of protection (14–16) and support the notion that intracellular parasite Ags, presumably expressed during LS development, are targets of immunity.

Using transcriptome data of LS forms of Pf parasites, we identified genes that are over-transcribed in LS parasites compared with either blood stage or SS (17). Importantly, we demonstrated that some *P. berghei* (Pb) orthologs of those Pf genes significantly reduce Pb LS parasite burden (LPB) in C57Bl/6 (B6) mice immunized with plasmid DNA delivered by the Gene Gun. The immune mechanisms that mediate LPB reduction are not fully understood, but in some instances are dependent on CD8 T cells, as depletion of CD8 T cells prior to spz challenge limits LPB reduction. We also showed that immunization with Pb or *P. yoelii* (Py) CSP combined with select LS Ags enhances protection in comparison with CSP alone (17). In the current study, we set out to identify MHC class I-restricted epitopes derived from the novel Pb LS Ags and to evaluate if such epitopes might contribute to protective immunity against spz challenge.

Given the size of the Plasmodium genome and the expression of unique antigenic proteins at each stage, it is not surprising that immunodominant epitopes for CD8 T cells have been difficult to identify. Detection of Ag-specific CD8 T cells became more direct and less laborious with the development of caged MHC-tetramer technology (18, 19). Briefly, this method is based on the design of conditional MHC class I ligands that form stable complexes with MHC molecules but degrade under UV light. This generates “empty” MHC molecules that can be loaded with other peptides. The newly formed MHC:peptide complexes can be used to monitor MHC class I-restricted peptide-specific CD8 T cell responses. This technology has been used to identify new MHC class I-restricted epitopes from viruses (20, 21), bacteria (22), and parasites (23, 24). We hypothesized that application of caged MHC class I-tetramer technology might uncover LS-derived epitopes specific for CD8 T cells involved in protection against malaria.

We have now identified a CD8 T cell epitope, Kb-17, contained within one of our recently described novel protective Pb LS Ags (17). CD8 T cells specific for Kb-17, identified by the caged tetramer technology, were detectable in mice immunized with Pb RAS and recalled following Pb spz challenge. Importantly, immune responses induced with Kb-17 conferred protection characterized by the reduction of Pb LPB. Identification of Kb-17 as a protective epitope not only underscores the role of LS Ag-specific CD8 T cell responses in protective immunity against LS parasites, but also establishes these responses as correlates of protection against malaria infection in the future.

## Materials and Methods

### Ethics Statement

All procedures were reviewed and approved by WRAIR/NMRC Institutional Animal Care and Use Committee (protocol # 15-MVD-30) and were performed in a facility accredited by the Association for Assessment and Accreditation of Laboratory Animal Care International in compliance with the Animal Welfare Act and in accordance with the principles set forth in the “Guide for the Care and Use of Laboratory Animals,” Institute of Laboratory Animals Resources, National Research Council, National Academy Press, 2011.

### Parasites

*Plasmodium berghei* (ANKA strain) (25) and luciferase-expressing Pb (Pb-luc) (ANKA strain) (26) spz were maintained by cyclical transmission in mice and *Anopheles stephensi* mosquitoes. Briefly, spz were dissected from the salivary glands of mosquitoes 17–21 days after an infective blood meal, as described previously (27). For challenge and immunization, spz were counted microscopically, adjusted to a given concentration in RPMI 1640 (Life Technologies, Grand Island, NY, USA) with 1% normal mouse serum, and used immediately after dissection to ensure maximal infectivity.

### Adenoviral and Plasmid DNA Constructs

Adenovirus serotype 5 (Ad5)-Kb-17 vector expressing the Kb-17 minigene (IVSFSFQNM) from PBANKA_1031000 with an HCMV promoter was purchased from Vector BioLabs (Malvern, PA, USA) (28). Plasmid DNA expressing PBANKA_1031000 gene and pCI plasmid empty vector (EV) were purchased from GenScript (Piscataway, NJ, USA).

### Mice

Six- to 8-week old female C57Bl/6J (B6) and C57BL/6-Tg (TcraTcrb) 1100Mjb/J (OT-1) mice were purchased from The Jackson Laboratory (Bar Harbor, ME, USA). Six- to 8-week old female C57BL/6NTac-*Tyr^tm1Arte^* (albino-B6) and B6.Cg-*Tcra^tm1Mom^*Tg (TcrLCMV)327Sdz (P14) mice were purchased from Charles River Laboratories (Wilmington, MA, USA). Animals were housed under specific-pathogen-free conditions at the Walter Reed Army Institute of Research animal facility and handled according to institutional guidelines. Autoclaved food and water were provided *ad libitum*.

### Immunizations and Challenge

**Figure 1:**
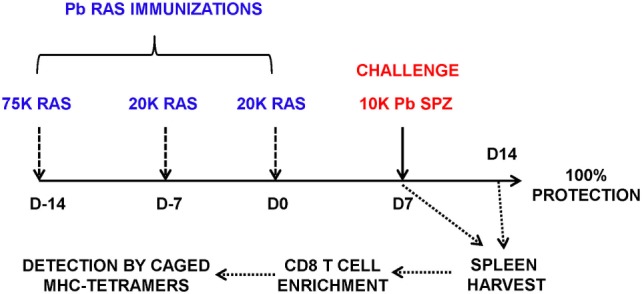
Schema of screening of CD8-restricted epitopes from *Plasmodium berghei* (Pb) liver stage (LS) antigens (Ags). B6 mice were immunized weekly three times with Pb radiation-attenuated sporozoite (RAS) intravenously (75, 20, and 20 K) and challenged 7 days after the last boost immunization with 10 K infectious Pb sporozoites (spz). Splenocytes were harvested at day 7 postchallenge, enriched for CD8 T cells using negative selection on magnetic microbeads, and stained with PE-labeled H-2K^b^ and H-2D^b^ tetramers exchanged with peptides from Pb LS Ags.

For immunizations, Pb spz were attenuated by γ-irradiation (15,000 rad) using a cobalt-60 source (Cobalt-60 Model 109; JL Shepard & Associates, San Fernando, CA, USA). Mice were primed intravenously (i.v.) with 75 K Pb RAS followed by two homologous boost immunizations of 20 K RAS given 1 week apart (Figure 1), unless otherwise noted. At indicated time points after the last Pb RAS immunization, mice were challenged i.v. with 10 K infectious Pb spz or Pb-luc spz.

For protection studies, B6 mice were immunized with plasmid DNA and Ad5 vector with a 6-week interval. Plasmid DNA (100 µg/100 μl PBS) was delivered intramuscularly into anterior tibialis muscles (50 µl per leg). Ad5 vector was delivered by i.v. injection into the tail vein at 2 × 10^9^ infectious units (ifu) followed by i.v. challenge with 10 K Pb spz or Pb-luc spz.

### Epitope Prediction for Pb LS Ags

Sequences of 32 proteins expressed by Pb ANKA (Table S1 in Supplementary Material) were extracted from PlasmoDB Plasmodium Genomics Resource database.^1^ The 32 selected open reading frames were analyzed by use of the Web-based predictive algorithms: BIMAS (29),^2^ RANKPEP (30),^3^ IEDB (31),^4^ and SYFPEITHI (32).^5^ Predicted nine amino acid residue epitopes with the highest integrative scores (see Results) were synthesized and purchased from Alpha Diagnostics International (Owings Mills, MD, USA). Lyophilized peptides were reconstituted in DMSO at a concentration of 10 mg/ml and stored at −80°C.

### MHC Class I Expression and Purification

Following established protocols (33), murine β_2_m and MHC class I H-2K^b^ and H-2D^b^ heavy chains that encode a BirA recognition sequence at the C-terminus (33, 34) were produced by recombinant expression in *Escherichia coli* strain BL21 induced with isopropyl β-d-thiogalactoside (IPTG). Inclusion bodies were purified, and the individual heavy chains were refolded with β_2_m and the conditional peptide SV9-P7* (22). The caged complexes were buffer-exchanged, and biotinylated using BirA biotin-protein ligase kit according to the manufacturer’s instructions (Avidity, Aurora, CO, USA). The H-2K^b^ and H-2D^b^ monomeric complexes were then purified by gel-filtration chromatography on a Sephadex S75 column (GE Healthcare Life Sciences, Little Chalfont, United Kingdom) and stored at −80°C.

### Multimerization and MHC Class I Peptide Exchange

Streptavidin-PE (Life Technologies) was added three times at 30 min intervals to biotinylated H-2K^b^ or H-2D^b^ monomers to a final molar ratio of 4:1 for monomer:Streptavidin-PE. Tetramers were stored at 4°C and used within 1 month. Tetramers were diluted to 50 µg/ml in DMEM without phenol red (Life Technologies) and supplemented with 10% fetal bovine serum (FBS) (Hyclone, South Logan, UT, USA), 1% Penicillin/Streptomycin (Gibco, Grand Island, NY, USA), 1% GlutaMAX (Gibco), and 0.1% NaN_3_ (Sigma, St. Louis, MO, USA). The solution was deposited in a 96-well plate (V-bottom, 110 µl per well), and index peptide was added (2 µl of 10 mM peptide per well). The plate was placed on ice and irradiated for 30 min in a CL-100 UV Crosslinker (UVP, Upland, CA, USA) equipped with 365 nm UV-lamps at a ≈10–20 cm distance. After 1 h of incubation at 37°C, the plate was centrifuged (3,000 × *g*) for 20 min, and 50 µl of the supernatant was transferred into new 96-well plate and used for cell surface staining (see Tetramer Staining and Flow Cytometry).

### Intrahepatic Mononuclear Cells (IHMC) Isolation

At the indicated time points after immunization or challenge mice were euthanized by CO_2_ inhalation. Livers were perfused with 10 ml PBS, removed and ground through 70 µM nylon cell strainer (BD Labware, Franklin Lakes, NJ, USA), and the cell suspension was processed as previously described (13). Briefly, cells were resuspended in PBS containing 35% Percoll (Amersham Pharmacia Biotec, Uppsala, Sweden) and centrifuged at 2,000 rpm for 20 min at room temperature with no brakes applied after centrifugation is completed. Pellet was collected and red blood cells (RBC) were lysed with RBC lysis buffer (Sigma) and the remaining IHMC were resuspended in complete RPMI 1640 medium containing 10% heat-inactivated FBS (Hyclone), 1% Penicillin/Streptomycin, 1% GlutaMAX, 1% HEPES, 1% non-essential amino acids (Gibco), 50 µM 2-mercaptoethanol (Sigma).

### Enrichment of Splenic CD8 T Cells

Spleens were harvested from mice and single cell suspensions were prepared from RBC-lysed splenocytes. CD8 T cells were isolated by negative selection using mouse CD8 T cell isolation kit by following the manufacturer’s protocol (Miltenyi Biotec, Auburn, CA, USA). Briefly, splenocytes were incubated with cocktail of biotinylated mAb (anti-CD4, -CD11b, -CD11c, -CD19, -CD45R, CD49b, CD105, -MHC class II, -Ter-119, and -TCR γ/δ) for 15 min at 4°C. Following washing in buffer (PBS containing 2 mM EDTA and 0.5% BSA), the cells were magnetically labeled with anti-biotin microbeads for 15 min at 4°C. The non-labeled cells were isolated by negative selection on LS columns according to the manufacturer’s instructions. The purity of the CD8 T cells was >95% and viability was >97% determined by Trypan blue exclusion.

### Tetramer Staining and Flow Cytometry

The following anti-mouse antibodies (Abs) from BD Biosciences (San Jose, CA, USA) were used: FITC- or PerCP-Cy5.5-conjugated anti-CD3e (clone 145-2C11), PerCP- or BV786-conjugated anti-CD4 (clone GK1.5), APC-, Horizon V500- or PerCP-conjugated anti-CD8α (clone 53-6.7), FITC-conjugated anti-CD11a (clone 2D7), Alexa 700-conjugated anti-CD44 (clone IM7), and PE-Cy7-conjugated anti-CD62L (clone MEL-14). Live/Dead Fixable Dead Cell Stain Kit for UV excitation (Life Technologies) was used to exclude dead cells. IHMC and enriched splenocytes were distributed among v-bottom 96-well plates (~1 × 10^5^–1 × 10^6^ cells in 50 µl per well) that contained saturating amounts of freshly prepared PE-labeled MHC tetramer and fluorochrome conjugated Abs listed above and were incubated for 45 min on ice. The cells were washed with PBS and fixed with 4% formaldehyde in PBS. Gating strategy is shown at Figure S1A in Supplementary Material. Flow cytometry was performed using LSRII system (BD Biosciences) and data were analyzed by FlowJo (v. 9.9.3, Tree Star Inc., Ashland, OR, USA) software.

For intracellular cytokine staining, cells were stimulated with 1 µg/ml of Kb-17 peptide for 14 h at 37°C; GolgiPlug (BD Biosciences) was added for the last 12 h of incubation. Cells were then stained for surface markers, fixed and permeabilized with BD Fix/Perm buffer and stained in Perm/Wash buffer with APC conjugated anti-IFN-γ Ab (clone XMG1.2) according to manufacturer’s instructions (BD Biosciences).

### RMA/S Stabilization Assay

RMA/S cells (35, 36) were grown in complete RPMI 1640 (see above) at 37°C. The day before experiment cells were plated at 10^6^ cells per well in a 24-well tissue culture plate and were transferred to 26°C overnight. Cells were pulsed with 9-mer peptides at the indicated concentrations for 30 min at 26°C followed by 3 h at 37°C. RMA/S cells were harvested, washed, and stained with FITC-conjugated anti-H-2K^b^ mAb (clone AF6-88.5, BD Biosciences) for 30 min on ice. Stained cells were washed, fixed with 4% formaldehyde, and analyzed by flow cytometry as described above. The results were computed as median of fluorescent intensity (MFI) of experimental samples pulsed with peptides divided by MFI of the media control sample.

### IFN-γ ELISPOT Assay

Liver and splenic lymphocytes were isolated, washed, and resuspended in complete RPMI medium (see above). BD ELISPOT plates were prepared using the mouse IFN-γ ELISPOT kit (BD Biosciences) according to the manufacturer’s instruction. Briefly, ELISPOT plates were coated with anti-IFN-γ Abs overnight at 4°C, subsequently washed with PBS and blocked using RPMI 1640 + 10% FBS for 2 h at room temperature and washed prior to use. Cells were plated into ELISPOT plates at a concentration of 200–300 × 10^3^ per well in 200 µl and stimulated with 1 µg/ml of Kb-17 (IVSFSFQNM) or Pb TRAP_130_ (SALLNVDNL) (12) peptides or medium alone for 42 h at 37°C. Plates were developed using Mouse IFN-γ ELISPOT Kit (BD Biosciences) according to manufacturer’s instructions. Results were quantified as the number of IFN-γ-specific spots per 10^6^ cells after subtracting results from medium control wells.

### Determination of LPB

Liver stage parasite burden was quantified by qPCR for Pb 18S ribosomal RNA (18S rRNA) as described previously (37). Total RNA was extracted from liver samples using Trizol according to manufacturer’s instructions. cDNA was synthesized using High-Capacity cDNA Reverse Transcription Kit (Applied Biosystems, Foster City, CA, USA). The qPCR reaction samples contained the following reagents in 25 µl volume: 12.5 µl of SYBR Green PCR Master Mix (Applied Biosystems), 0.1 µM of either Pb 18S rRNA primers (forward—5′-AAGCATTAAATAAAGCGAATACATCCTTAC-3′; reverse—5′-GGAGATTGGTTTTGACGTTTATGTG-3′) or mouse β-actin gene (forward—5′-GGCTGTATTCCCCTCCAT-3′; reverse—5′-CCAGTTGGTAACAATGCAAT-3′) and 2 µl of 1:10 dilution of cDNA sample. The reaction was run on 7500 Fast qPCR System (Applied Biosystems) using the following conditions: 15 min at 95°C, 40 cycles with 95°C for 20 s; 60°C for 30 s, and 72°C for 50 s.

cDNA standards were prepared as 10-fold serial dilutions of purified PCR products for both 18S rRNA and β-actin from 10^8^ to 10^5^ copies per 2 µl. Each reaction was set up in triplicate. Livers of naive mice served as a negative control. Parasite load was calculated as ratio of 18S rRNA to host β-actin (housekeeping gene) expression. Protection was defined as a statistically significant reduction of parasite burden in the livers of experimental mice compared with mice immunized with EV.

### *In Vivo* Imaging System (IVIS)

*In vivo* imaging studies of bioluminescence activity from Pb-luc infected mice were performed using an IVIS Spectrum System (Perkin Elmer, Hanover, MD, USA), as described previously (38). LPB was detected at 24 and 48 h post challenge. Mice received 150 mg/kg of luciferin (Gold Biotechnology, St. Louis, MO, USA) i.p. in 200 µl of PBS. Three minutes post luciferin administration, mice were anesthetized by isoflurane inhalation. Mice were then positioned ventral side up in the IVIS chamber on a 37°C heating platform and continued to receive isoflurane through nose cones. Exposure time was set to 5 min or until complete saturation with f-stop = 1 and large binning setting. Bioluminescence emitted from the region of intensity (ROI) was measured as luminescence signal intensity in photons per second using the ROI settings of the Living Image^®^ 3.0 software.

### Statistical Analysis

For experimental data presented in this manuscript, Shapiro–Wilk normality test was performed prior to statistical analysis and no significant departures from normality were detected. Significant difference between the data points was compared by ordinary one-way ANOVA test followed by Tukey’s multiple comparisons test, two-way ANOVA test followed by Sidak’s multiple comparisons test, or Kruskal–Wallis test followed by Dunn’s multiple comparisons test. Values of *p* < 0.05 were considered as significant. Statistical data analysis was performed using GraphPad Prism version 6.07 for Windows (GraphPad Software, San Diego, CA. USA).

## Results

### Epitope Prediction for Novel Pb LS Ags

On the basis of recently published results demonstrating protective and non-protective novel Plasmodium LS Ags (17), we selected a panel of 32 Pb LS Ags, and utilizing four Web-based predictive algorithms, we searched protein sequences for the presence of H-2^b^-restricted nonameric epitopes. We ranked peptides based on their scores in BIMAS, RANKPEP, IEDB algorithms for H-2K^b^ and BIMAS, RANKPEP, IEDB, SYFPEITHI algorithms for the H-2D^b^-molecule. Overall rank was calculated as a sum of all ranks for each predicted peptide. The cut-off limit was set as a rank <372 and <955 for Kb and Db algorithms, respectively. This rank calculation resulted in 142 H-2K^b^- (Table S2 in Supplementary Material) and 254 H-2D^b^- (Table S3 in Supplementary Material) restricted unique candidate sequences. A total of 396 9-mer peptides were subsequently synthesized and then used to generate distinct caged MHC class I tetramers.

### Screening for CD8 T Cell Epitopes from Pb LS Ags

**Figure 2:**
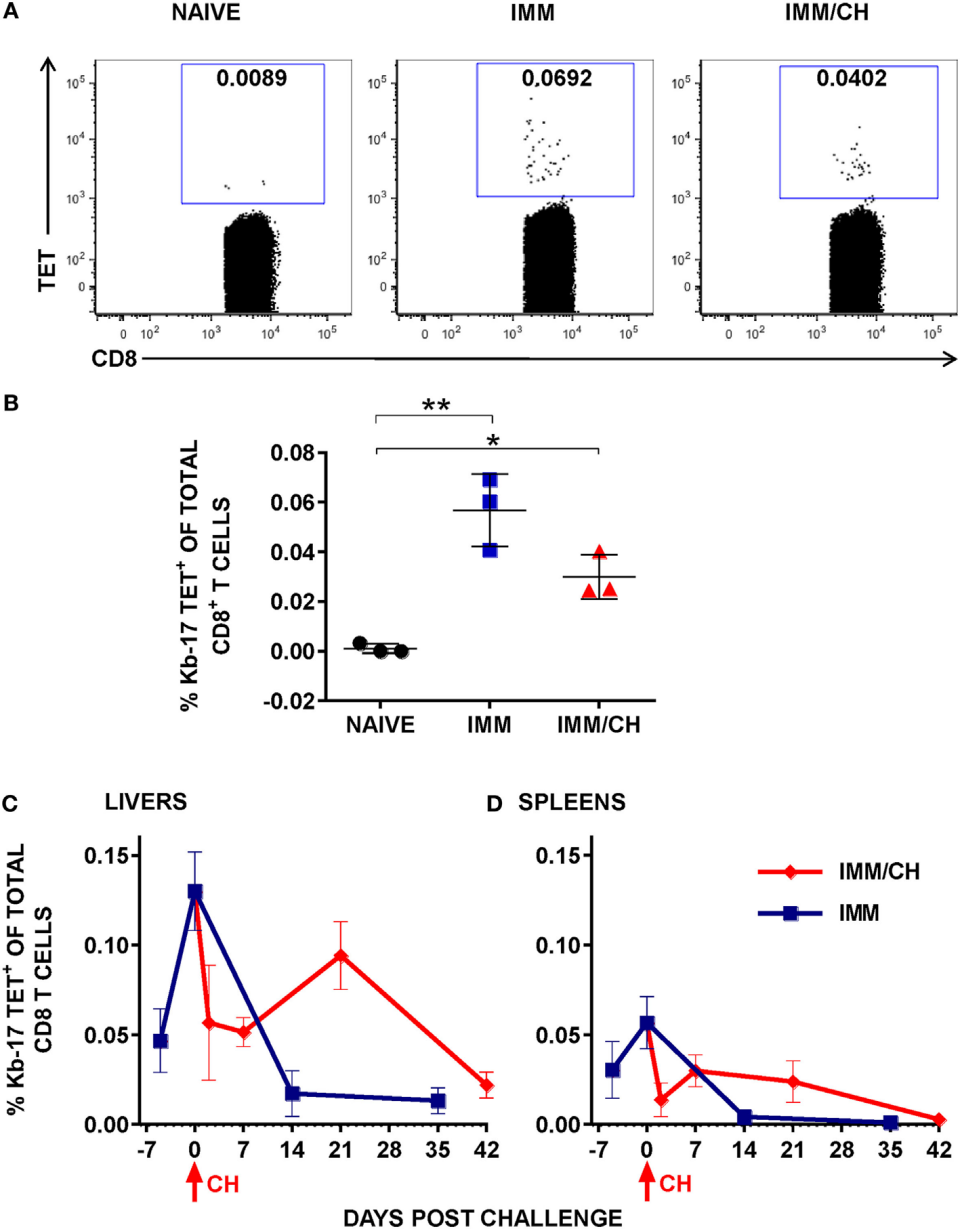
Immunization with *Plasmodium berghei* (Pb) radiation-attenuated sporozoite (RAS) and challenge with Pb sporozoites (spz) induced expansion of Kb-17-Tet^+^CD8 T cells. **(A,B)** B6 mice were immunized and challenged as described in Figure 1. Enriched splenic CD8 T cells from naïve, RAS immunized (IMM), and immunized/challenged (IMM/CH) mice were stained with PE-labeled H-2K^b^-tetramers exchanged with the Kb-17 peptide (PBANKA_103100). **(A)** Representative data displayed are from single mouse and values represent the percentage of Kb-17-Tet^+^CD8 cells from total CD3^+^CD8^+^ population. **(B)** Representative results from one out of four experiments expressed as percentage of Kb-17-Tet^+^ CD8 T cells of total splenic CD3^+^CD8^+^ T cells and are shown individual mice with lines representing the mean and error bars indicating standard deviation. *p < 0.05; **p < 0.01; ordinary one-way ANOVA test followed by Tukey’s multiple comparisons test. **(C,D)** Mice immunized thrice with Pb RAS were challenged with10 K infectious Pb spz intravenously 7 days after the last boost immunization or were left unchallenged. Kinetics of **(C)** liver and **(D)** spleen Kb-17-Tet^+^ CD8 T cells were analyzed at the various time points, including the day of last boost immunization with Pb RAS (day −7), day of challenge (day 0), and the indicated time points thereafter. Representative results from one out of two experiments expressed as percentage of Kb-17-Tet^+^CD8 T cells of total liver or splenic CD8 T cells are shown as the mean of three individual mice per group, per time point.

It has been well established that in a mouse model of protective immunity induced with RAS, CD8 T cells mediate protection against experimental spz challenge (7). We also demonstrated that the protective CD8 T cells are MHC class I-dependent and that they recognize Pb Ags derived from the LS parasites (8). The discovery of novel LS Ags that are targets of protection against spz challenge prompted us to ask if epitopes found on the protective LS Ags could be identified following Pb RAS immunization so that protective CD8 T cells could be traced during the induction and/or the effector phase of protection. To identify novel Pb LS Ag-specific CD8 T cell epitopes, MHC class I tetramers were prepared from 396 Pb predicted epitopes and photocleavable H-2K^b^/SV9-P7* or H-2D^b^/SV9-P7* complexes by UV-mediated exchange (22). Enriched CD8 T cells from spleens of naïve, thrice Pb RAS immunized, and Pb RAS-immunized/Pb spz challenged B6 mice (Figure 1) were incubated with tetramers representing putative Ags and binding was analyzed by flow cytometry staining. As positive controls for H-2K^b^ and H-2D^b^ tetramer staining, we used splenocytes from OT-1 and P14 mice that carry TCR specific for CD8 T cell epitopes from chicken ovalbumin (OVA_257–264_) and LCMV glycoprotein (gp33), respectively (Figure S1B in Supplementary Material). Enriched CD8 T cells from naïve mice served as negative controls. We found one H-2K^b^-restricted epitope with the sequence IVSFSFQNM (Kb-17) from PBANKA_1031000 and the tetramer Kb-17 (Kb-17-Tet) recognized splenic CD8 T cells from mice immunized with Pb RAS or Pb RAS-immunized/Pb spz challenged mice (Figures 2A,B). Interestingly, the Kb-17 peptide is derived from Pb LS gene, PBANKA_1031000, which as plasmid DNA vaccine protects B6 mice (17). The binding capacity of Kb-17 to H-2K^b^ molecule measured as an ability to rescue MHC class I molecules was validated by RMA/S cell stabilization assay (Figure S2A in Supplementary Material). In order to test the specificity of binding to H-2K^b^ molecule, we demonstrated that Kb-17-tetramer did not stain control splenocytes from transgenic OT-1 mice (Figure S2B in Supplementary Material).

### Kinetics of Kb-17-Tet^+^ CD8 T Cells and IFN-γ Responses

Persistence of memory CD8 T cells in the liver and their recall upon infectious spz challenge is the key feature of long-lasting protection in animal models induced with Pb RAS (13, 39). In the next series of experiments, we conducted analyses of the frequency and kinetics of Kb-17-specific CD8 T cell responses in Pb RAS-immunized/Pb spz challenged mice. IHMC and splenic cells were stained with the Kb-17-Tet at the indicated time points that include day 7 after the last Pb RAS boost immunization (day −7), the day of challenge (day 0), as well as several time points after the challenge (Figures 2C,D). In the livers of Pb RAS-immunized mice, the Kb-17-Tet^+^CD8 T cells increased from 0.05% on day −7 to ~0.15% of the total liver CD8 T cells on day 0, whereas in the spleens the Kb-17-Tet^+^CD8 T cells increased from 0.03% at day −7 to 0.05% of CD8 T cells on the day 0.

Determinations of Kb-17-Tet^+^ CD8 T cells in the group of Pb RAS-immunized/Pb spz challenged mice showed that the percentage of these cells declined precipitously at 2 days post challenge in both the liver and the spleen. In the liver, the Kb-17-Tet^+^ CD8 T cell levels returned to nearly 0.1% at 3 weeks after the challenge (day 21), but at 6 weeks (day 42), the level declined to ~0.025%. In the spleens the percentage of the Kb-17-Tet^+^ CD8 T cells rebound to 0.025% at 1 week (day 7) after the challenge but by 6 weeks after challenge (day 42), Kb-17-Tet^+^ CD8 T cells were undetectable. A much faster decline of the Kb-17-Tet^+^CD8 T cells was observed in the liver and the spleens of mice that were not challenged; the cells returned to a very low level (<0.01) in the livers and in the spleens they were nearly undetectable on days 14 and 35, time points which are equivalent to days 21 and 42 after the last Pb RAS boost immunization.

**Figure 3:**
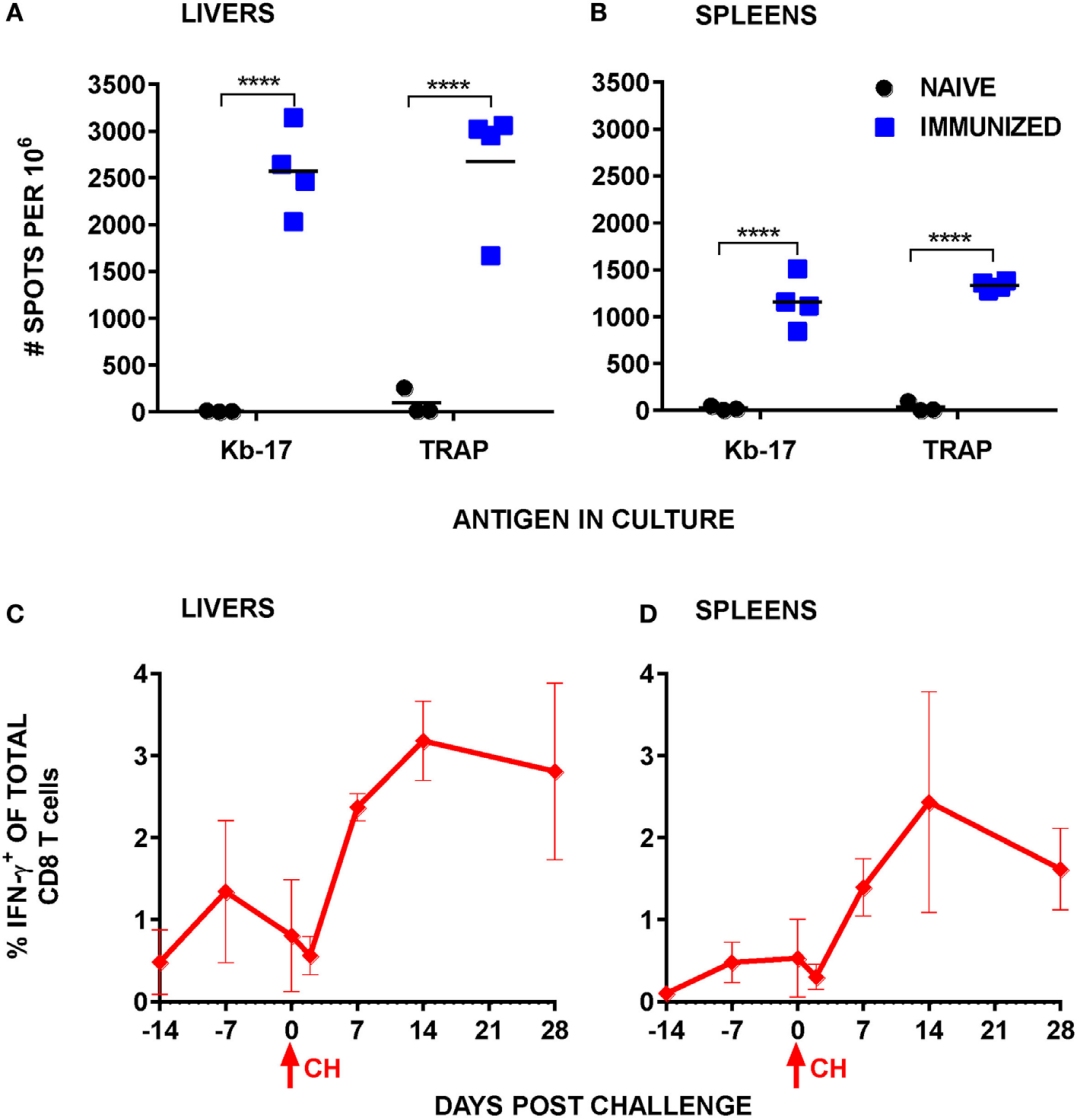
Stimulation with Kb-17 peptide induces production of IFN-γ by intrahepatic mononuclear cells (IHMC) and splenocytes from mice immunized with *Plasmodium berghei* (Pb) radiation-attenuated sporozoite (RAS). **(A,B)** IHMC and splenocytes, isolated from naïve and Pb RAS-immunized mice according to the timeline shown in Figure 1, were stimulated with Kb-17 peptide *in vitro* in anti-IFN-γ coated ELISPOT plates for 42 h. Unstimulated cells served as a negative control; cells stimulated with PbTRAP_130_ peptide served as a positive control. Representative results from two combined experiments are expressed as the net number of IFN-γ^+^ spots per 10^6^ cells following subtraction of spots from unstimulated control wells. ****p < 0.0001; two-way ANOVA test followed by Sidak’s multiple comparisons test. **(C,D)** B6 mice were immunized by intravenously injection with 75 K Pb RAS followed by two boosts with 20 K RAS every 2 weeks and challenged with 10 K Pb sporozoites 2 weeks after the last boost. Liver and spleen cells were intracellularly stained for IFN-γ at the indicated time points. Day −14 represents the day of the third immunization, day −7 represents 7 days after the third RAS immunization, and day 0 is the day of challenge. Results are expressed as percentage of IFN-γ^+^ cells of total CD3^+^CD8^+^ T cells in the liver or the spleen. Results are representative of one out of two experiments and shown as the mean of three individual mice per group/per time point. Arrows indicate the day of challenge.

According to the majority of published studies, IFN-γ plays a significant role in eliminating LS forms of malaria parasites (40), be it indirectly or during a contact between the effector CD8 T cells producing the cytokine and the infected hepatocytes (41). Apart from demonstrating that the numbers of Kb-17-Tet^+^ CD8 T cells increased following Pb RAS immunization and spz challenge, we also tested the induction of IFN-γ responses in spleens and IHMC stimulated with the Kb-17 peptide. According to our results, Kb-17 recalled specific IFN-γ responses amongst mononuclear cells in both organs of Pb RAS-immunized mice, but not naïve B6 mice (Figures 3A,B). The magnitude of responses was comparable with a response recalled with the previously described protective H-2D^b^ restricted Pb TRAP_130_ peptide (12).

We also quantified Kb-17-recalled IFN-γ^+^ CD8 T cell responses at several time points after Pb RAS immunization/Pb spz challenge. Overall, responses in both the liver and the spleen (Figures 3C,D) increased from undetectable levels in naïve mice to ~1.5% of total liver CD8 T cells and ~0.5% of total splenic CD8 T cells after the last boost immunization. Similar to the tetramer kinetics results, IFN-γ^+^CD8 T cells declined in both organs immediately after the challenge, but increased to ~3% in the liver and ~2.5% in the spleen.

### Immunization with Kb-17 as Ad5 Minigene Reduces LPB

**Figure 4:**
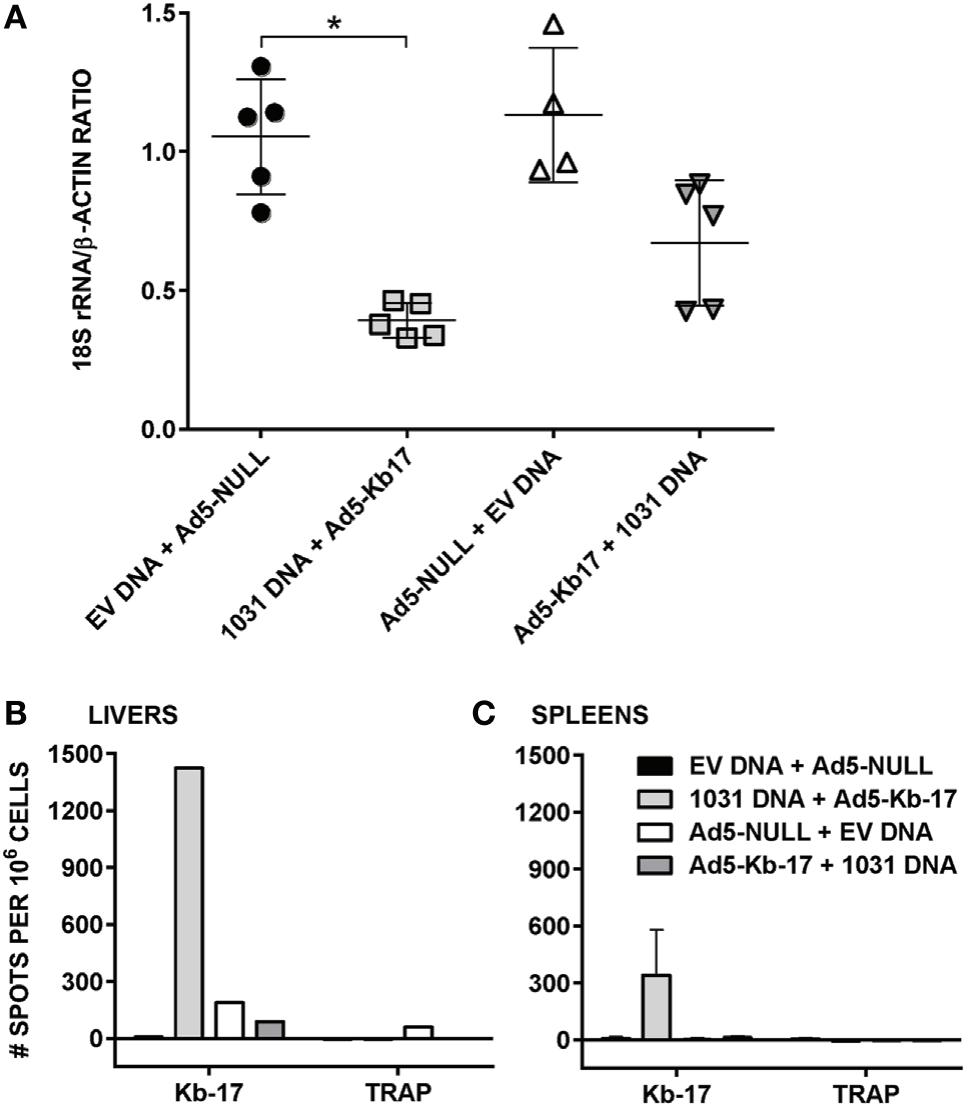
Immunization with adenovirus serotype 5 (Ad5)-Kb-17 minigene vector reduces liver stage parasite burden (LPB) after *Plasmodium berghei* (Pb) sporozoites (spz) challenge and induces CD8 IFN-γ responses in the spleens and livers. **(A)** B6 mice were primed intramuscularly with 100 µg PBANKA_103100 plasmid DNA and boosted intravenously (i.v.) with 2 × 10^9^ infectious units Ad5-Kb-17 vector 6 weeks after the prime (or vice versa) and challenged with 10 K Pb spz i.v. Reduction of LPB was determined by qPCR in the livers 40 h after the challenge and expressed as ratio of Pb 18S rRNA to mouse β-actin. Immunization with empty plasmid DNA [empty vector (EV) DNA] and empty Ad5 (Ad5-null) served as a negative control. Results representing one out of two experiments are presented. *p < 0.01; Kruskal–Wallis test followed by Dunn’s multiple comparisons test. **(B,C)** Intrahepatic mononuclear cells and splenocytes were harvested 7 days after the boost immunization and stimulated with Kb-17 peptide *in vitro* in ELISPOT plates for 42 h. Non-stimulated cells and cells stimulated with PbTRAP_130_ peptide served as a negative control. Representative results of one out of three experiments are shown for pooled livers from three mice **(B)** or as the mean of three individual mice per group **(C)** and expressed as the net number of IFN-γ^+^ spots per million cells after subtraction of unstimulated samples.

Vaccination with vaccinia virus expressing CD8 T cell epitopes as short minigenes can partially protect mice against viral and bacterial infections (42, 43). Because the Kb-17-recalled IFN-γ^+^ CD8 T cell responses and Kb-17-Tet^+^ CD8 T cells rose after Pb RAS immunization and were recalled following challenge, we hypothesized that Kb-17-specific CD8 T cells may be involved in mediating, at least partially, protective immune responses to Pb spz challenge. We used the constructed plasmid DNA encoding PBANKA_1031000 gene (pCI-1031) and Ad5 expressing Kb-17 peptide as a minigene (Ad5-Kb-17) to test this hypothesis in our experimental model system. We compared two immunization schedules: DNA prime/Ad5 boost and Ad5 prime/DNA boost, delivering 100 µg of plasmid DNA and 2 × 10^9^ ifu of Ad5 i.v. with 6 weeks interval between prime and boost immunization regimen. To determine if this immunization strategy induced protection as defined by a reduction of LPB, mice were immunized as described above and challenged with 10 K Pb spz 14 days after the last boost and LPB was determined by qPCR at ~40 h after the challenge (Figure 4). Immunization of mice with pCI-1031 DNA and Ad5-Kb-17 significantly reduced LPB in B6 mice infected with Pb spz when compared with EV DNA/Ad5 null immunized control mice (Figure 4A). Priming with Ad5 and boosting with DNA also conferred protection, however, less effective, albeit not statistically significant. *In vitro* stimulation of IHMC and splenocytes from mice vaccinated with pCI-1031 DNA and Ad5-Kb-17 recalled robust IFN-γ production, whereas no IFN-γ responses were observed in mice vaccinated with Ad5-Kb-17 plus pCI-1031 DNA (Figure 4B,C). The absence of IFN-γ responses was surprising; one possibility is that the time lapse from the Ad5-Kb-17 priming to the time of T cell analyses caused the Kb-17-specific cells to contract to the point that were not recalled with the peptide. In this case, the DNA boost immunization was insufficient to induce and maintain the IFN-γ-producing Kb-17-specific T cells. Pb TRAP_130_ peptide stimulated cultures resulted in negative IFN-γ responses (Figure 4B,C). A boost immunization with Ad5-Kb-17 expanded the population of liver Kb-17-Tet^+^ CD8 T cells expressing CD11a^+^CD44^+^CD62L^lo^ phenotype, hence effector memory cells (Figure S3 in Supplementary Material).

**Figure 5:**
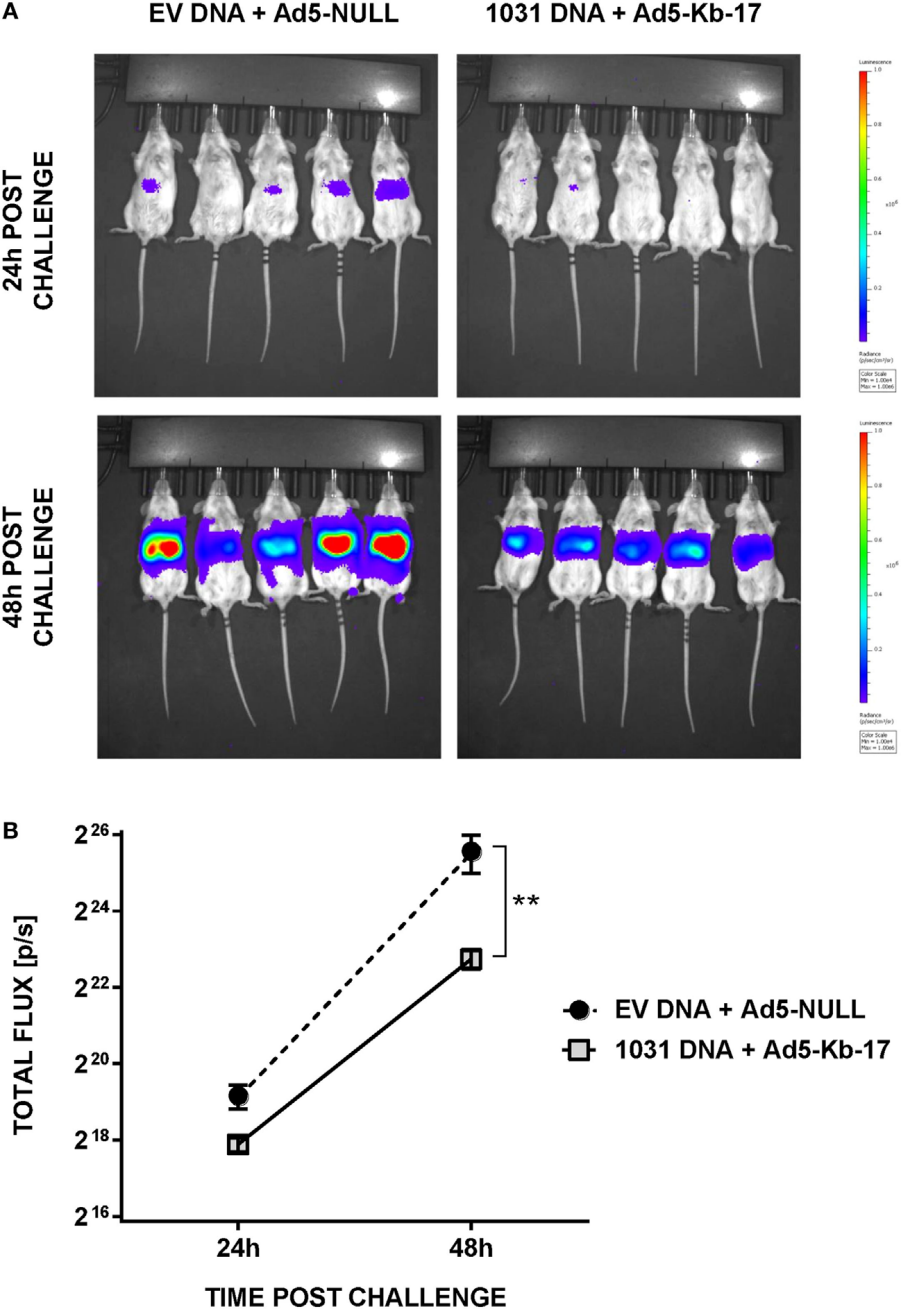
Immunization with DNA and adenovirus serotype 5 (Ad5)-Kb-17 induces reduction in liver stage parasite burden determined by *in vivo* imaging system. Albino-B6 mice were immunized by heterologous prime-boost with DNA and Ad5-Kb-17 at a 6-week interval as described in the legend for Figure 4 and challenged with 10 K Pb-luc sporozoites i.v. **(A)** Representative *in vivo* images of luminescence in the livers are shown at 24 and 48 h postchallenge. **(B)** Luminescence signal intensity in the livers at 24 and 48 h postchallenge. Representative results from one out of two experiments are shown as the mean of five individual mice per group. **p < 0.01; two-way ANOVA test followed by Sidak’s multiple comparisons test.

In addition to measuring LPB by qPCR, we used whole body IVIS to assess protection in albino-B6 mice infected with Pb-luc spz (38) (Figure 5). This approach allows an early detection, 24 and 48 h, of infection in treated and control animals (Figure 5A). Mean luminescence values (photon counts per second) collected from an ROI corresponding to the liver of mice immunized with EV DNA and Ad5-null were 2.4-fold higher at 24 h and 7.1-fold higher at 48-h postinfection compared with animals vaccinated with pCI-1031 DNA and Ad5-Kb-17 (Figure 5B). Therefore, these data are consistent with a protective role for Kb-17-specific CD8 T cells, which directly recognize Kb-17 expressed on target LS parasite infected cells following experimental spz challenge.

## Discussion

The discovery of antigenic targets of protective immunity has been the single most important goal of the entire malaria community interested in the development of anti-malaria vaccines. Different approaches, including *in silico* screening (44) and high-throughput adenovirus array (45), have been utilized to identify novel LS Ags. Recently, using transcriptome approach, we identified several LS Ags that confer partial protection against rodent malaria spz challenge and protection is enhanced by combining LS Ags with CSP (17). Protection induced by some LS Ags is mediated by CD8 T cells, but fine specificities of effector CD8 T cells remain for the most part unknown. Here, we report that by utilizing caged MHC class I-tetramer technology (18), we identified Pb LS Ag-derived H-2K^b^-restricted 9-mer epitope, Kb-17 (IVSFSFQNM). The Kb-17-tetramer specifically bound CD8 T cells from Pb RAS-immunized and Pb RAS-immunized/Pb spz challenged B6 mice. Notably, when expressed as a single epitope contained within an Ad5 vector, Kb-17-specific CD8 T cells mediated a reduction of LPB against spz challenge.

The Kb-17 peptide was predicted from the Pb LS Ag protein sequence of PBANKA_1031000, which—as a plasmid DNA vaccine—induces protection measured as a reduction of LPB in B6 mice exposed to Pb spz challenge (17). We also demonstrated that not only is the Kb-17 ancestor, PBANKA_1031000, one of the most protective Ags in the Pb-B6 experimental model, but its Py ortholog, PY00162, also confers partial protection mediated by CD8 T cells in Balb/c mice (17). Little is known about the function of the Pf ortholog, PF3D7_1411500. However, this gene contains motifs of the middle domain of eukaryotic initiation factor 4G (MIF4G), which is a component of the translation initiation factor eIF4F complex and has an important role in the initiation of mRNA translation (46). Apart from its presumed functional importance for the parasite, our results indicate that this Pb “MIF4G-like-protein” was expressed by Pb LS parasites derived from both Pb RAS as well as from Pb infectious spz. Clearly, this protein gained an entry into the MHC class I presentation pathways, as Kb-17 peptide recalled IFN-γ^+^ CD8 T cell responses and Kb-17-Tet^+^ CD8 T cells were detectable in spleens and livers from Pb RAS-immunized mice. Importantly, kinetics studies showed that Kb-17-Tet^+^ CD8 T cells were detected in the livers and spleens of Pb RAS-immunized/Pb spz challenged mice. This implies that the MIF4G-like protein was expressed during LS development following infection with spz and was processed by the TAP-dependent pathway. We have previously demonstrated that while LS Ags derived from Pb RAS could be processed by either or both TAP-dependent as well as TAP-independent pathways, the recall of effector CD8 T cells during challenge with infectious spz is TAP-dependent; unlike WT mice, Pb RAS-immunized TAP deficient mice are not protected and CD8 T cells remain unresponsive to infectious spz challenge, although CD8 T cells are activated by Pb RAS immunization (47). We therefore propose that the Pb MIF4G-like protein was available to enter Ag processing and presentation pathways and the resulting Kb-17 peptide either bound to nascent MHC class I molecules in the ER (48) or to the recycling MHC class I molecules in phagosomal-endosomal compartment of Ag presenting cell (49) was available for both the activation and recall of protective CD8 T cells.

The Kb-17-Tet^+^ CD8 T cells as well as IFN-γ^+^CD8 T cells induced by whole parasite RAS immunization declined sharply shortly after spz challenge. Likely, this resulted from a surge of inflammatory cytokines released in response to infectious spz. IFN-γ in particular enhances cellular attrition owing to apoptosis of effector CD8 T cells that show elevated expression of IFN-γR after infections with Pb spz (50). Both the level of Kb-17-Tet^+^ CD8 T cells as well as the Kb-17 recalled IFN-γ^+^CD8 T cells returned to pre-challenge levels at 2–3 weeks after the challenge. The ability of Kb-17-specific CD8 T cells to mediate a reduction of LPB in response to spz challenge underscores the importance of the LS Ags derived from spz. Collectively, these observations lend support to the notion that LS Ags do induce protective CD8 T cells and as such, these Ags should be considered as a strategy to be included with SS Ags, CSP, or TRAP, as a viable option for an improved malaria vaccine.

Based on the size of Plasmodium genome ~23 Mb (51), a large number of CD8 T cell epitopes should be available for binding to MHC class I molecules to form MHC:peptide complexes for activation of CD8 T cells. Despite many years of research devoted to finding epitopes of Ag-specific T cells involved in protective immunity, currently there are but a few known mouse MHC class I-restricted Pb epitopes derived from the PE stage that induce IFN-γ^+^ CD8 T cell responses and confer protective immunity: H-2^d^ restricted epitope PbCSP_253–261_ (52–54) and H-2^b^ restricted PbTRAP_130–138_ (12).

Multiple factors may account for the difficulties in finding more CD8 T cell epitopes among the many different proteins expressed during the entire PE stage and particularly the LS. The initial peptide selection for the tetramer formation was based on *in silico* determinations and this in itself contains inherent limitations, as only certain algorithm-dictated binders are selected to be included in the pool of epitopes for testing and no allowances are made for post-translationally modified epitopes. In accordance with this approach, many potential epitopes are rejected owing to hierarchical estimates of MHC class I binding scores. Similar to our findings with ~400 peptides assayed in the tetramer binding assay, others have shown that only two peptides, from a pool of 600 overlapping 8–10-mer peptides used for stimulations, recognize and activate CD8 T cells derived from immunization with RAS (12).

Alternatively, the abundance of proteins expressed during the LS (17) may give rise to a rather large number of peptides competing for binding to and occupancy of the MHC class I molecules in the ER or in other cellular compartments where a peptide exchange may be occurring. On the basis of the H-2^b^ binding indices, it appears that Pb LS Ag peptides display lower scores than PbCSP_253–261_ peptide. This may lead to a failure of many LS Ag peptides to associate with MHC or the formed MHC:peptide complexes may be unstable, which would preclude their export to the surface of APCs. According to this scenario, stable MHC class I:peptide complexes with a strong affinity for the TCR would be infrequent, which may explain the rather weak CD8 T cell responses that have been noted in previous studies describing responses to malaria Ags (55). In comparison with the percentage of tetramer positive CD8 T cells observed in viral (56) infections, the levels observed in our studies are rather low. The paucity of MHC class I epitopes detected from the LS parasites could also be attributed to the short duration of this stage. During infection, effector CD8 T cells must be recalled swiftly and therefore, effector CD8 T cells may recognize only the most abundant and most available MHC:peptide complexes on target cells. It is also possible that the Plasmodium parasite may have developed a strategy for avoiding generation of peptides by the virtue of not having easily accessible cleavage sites on the potentially antigenic proteins.

In summary, we identified a novel protective CD8 T cell epitope, Kb-17, from the protective LS Ag, PBANKA_1031000. We expect that the identification of this epitope will provide much needed information to advance our understanding of the requirements of specific CD8 T cells for the induction and maintenance of lasting protection against malaria and will serve as a tool to manipulate CD8 T cell responses to enhance protective immunity. Identification of PBANKA_1031000 as a target of protective CD8 T cell responses in the Pb-B6 model strongly supports testing of its Pf ortholog, PF3D7_1411500, as a potential vaccine candidate against malaria.

## Ethics Statement

All procedures were reviewed and approved by WRAIR/NMRC Institutional Animal Care and Use Committee (protocol # 15-MVD-30) and were performed in a facility accredited by the Association for Assessment and Accreditation of Laboratory Animal Care International in compliance with the Animal Welfare Act and in accordance with the principles set forth in the “Guide for the Care and Use of Laboratory Animals,” Institute of Laboratory Animals Resources, National Research Council, National Academy Press, 2011.

## Author Contributions

AP and UK conception or design of the work. AP, SZ, and LP acquisition and analysis of data. AP, SZ, PD, HP, and UK interpretation of data for the work. AP, SZ, and UK drafting the work. AP, SZ, LP, PD, HP, and UK revising the work critically for important intellectual content. AP, SZ, LP, PD, HP, and UK final approval of the version to be published. AP, SZ, LP, PD, HP, and UK agreement to be accountable for all aspects of the work in ensuring that questions related to the accuracy or integrity of any part of the work are appropriately investigated and resolved.

## Conflict of Interest Statement

The authors declare that the research was conducted in the absence of any commercial or financial relationships that could be construed as a potential conflict of interest.

## Footnotes

^1^www.plasmodb.org.

^2^http://www-bimas.cit.nih.gov/molbio/hla_bind/.

^3^http://bio.dfci.harvard.edu/RANKPEP.

^4^www.iedb.org.

^5^www.syfpeithi.de.

## Supplementary Material

Figure S1 Gating strategy for detection of Kb-17-Tet^+^ cells and staining of splenocytes from transgenic mice with control caged MHC-tetramers. **(A)** Lymphocytes are first identified by forward (FSC) and side (SSC) scatter followed by dead cell exclusion based on Live/Dead Fixable Dead Cell Stain uptake; single cells are identified by FSC-A and FSC-H; and finally live single lymphocytes are analyzed for their CD3 and CD8 expression. **(B)** Splenocytes from transgenic OT-1 and P14 mice were stained with APC-labeled anti-CD8 mAbs and tetramers were generated using UV peptide exchange PE-labeled H-2K^b^ and H-2D^b^ tetramers with OVA_257–264_ (SIINFEKL) and gp33 (KAVYNFATC) peptides, respectively.

Figure S2 Gating strategy for detection of Kb-17-Tet+ cells and staining of splenocytes from transgenic mice with control caged MHC-tetramers. **(A)** Lymphocytes are first identified by forward (FSC) and side (SSC) scatter followed by dead cell exclusion based on Live/Dead Fixable Dead Cell Stain uptake; single cells are identified by FSC-A and FSC-H; and finally live single lymphocytes are analyzed for their CD3 and CD8 expression. **(B)** Splenocytes from transgenic OT-1 and P14 mice were stained with APC-labeled anti-CD8 mAbs and tetramers were generated using UV peptide exchange PE-labeled H-2Kb and H-2Db tetramers with OVA257-264 (SIINFEKL) and gp33 (KAVYNFATC) peptides, respectively.

Figure S3 Immunization with Ad5-Kb-17 minigene vector expands Kb-17 specific CD8 T cells in the livers. Kb-17 tetramer positive liver CD8^+^ T cells (panel 1) harvested 7 days postboost have phenotype of antigen experienced CD11a^+^ (panel 2) CD44^+^CD62L^−^ (panel 3) effector memory CD8 T cells (blue dots on the panels 2 and 3 represent tetramer-positive CD8^+^ T cells). The numbers represent percentage of tetramer-positive cells of total CD8 T cells (panel 1) and proportions of tetramer positive cells inside the corresponding gates among total tetramer-positive cells (panels 2 and 3).
