# Supplementary figures and images for: Identification of a Novel CD8 T Cell Epitope Derived from Plasmodium berghei Protective Liver-Stage Antigen
Source: Front Immunol. 2018 Jan 29;9:91. doi: 10.3389/fimmu.2018.00091 (PMC5796907; doi:10.3389/fimmu.2018.00091)

**(A)**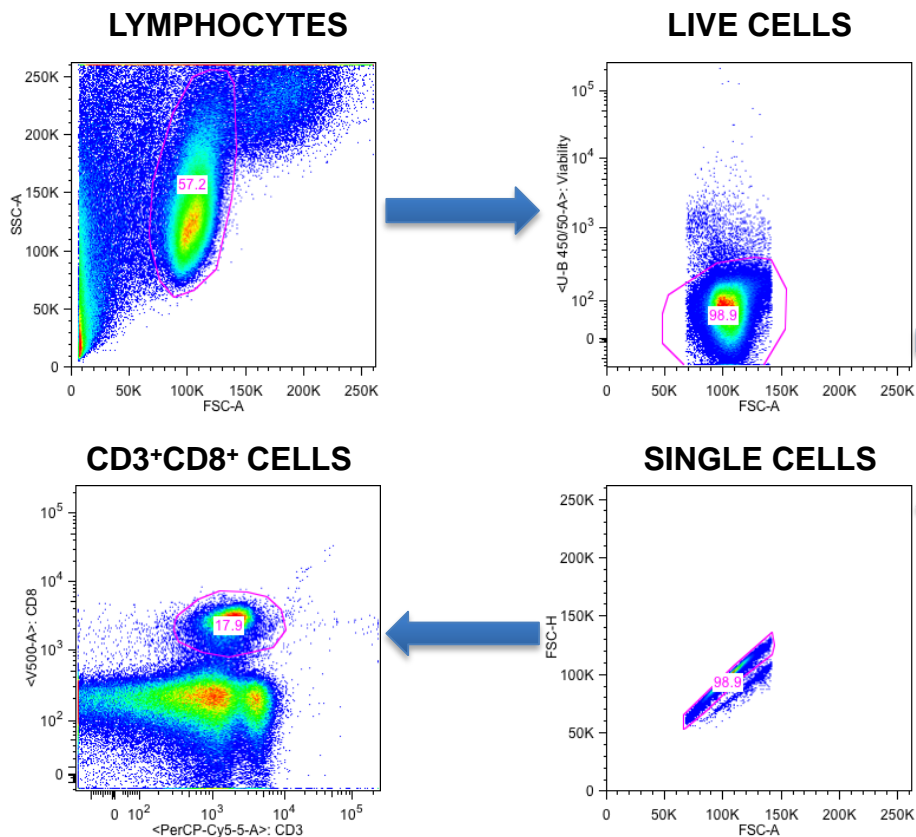**(B)**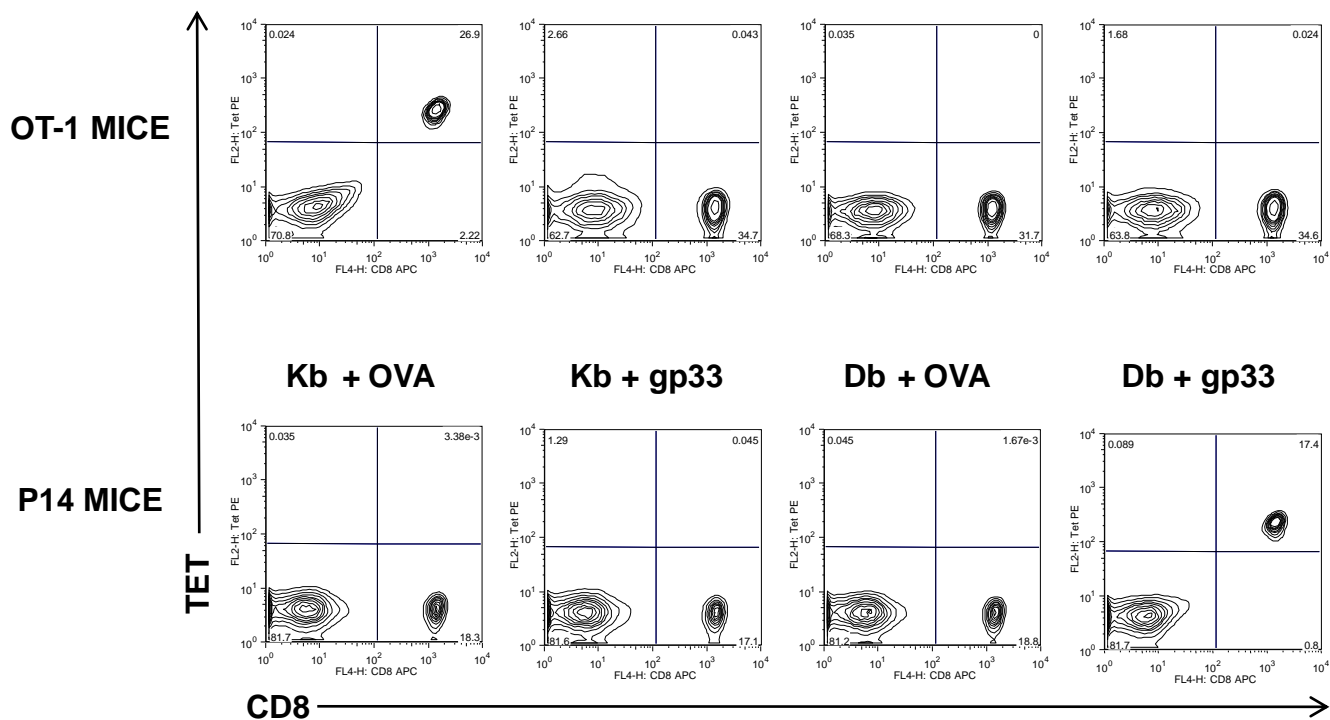**Figure S1**

Supplement: Figure S1 — Gating strategy for detection of Kb-17-Tet+ cells and staining of splenocytes from transgenic mice with control caged MHC-tetramers. (A) Lymphocytes are first identified by forward (FSC) and side (SSC) scatter followed by dead cell exclusion based on Live/Dead Fixable Dead Cell Stain uptake; single cells are identified by FSC-A and FSC-H; and finally live single lymphocytes are analyzed for their CD3 and CD8 expression. (B) Splenocytes from transgenic OT-1 and P14 mice were stained with APC-labeled anti-CD8 mAbs and tetramers were generated using UV peptide exchange PE-labeled H-2Kb and H-2Db tetramers with OVA257–264 (SIINFEKL) and gp33 (KAVYNFATC) peptides, respectively. [file image_1.PDF]

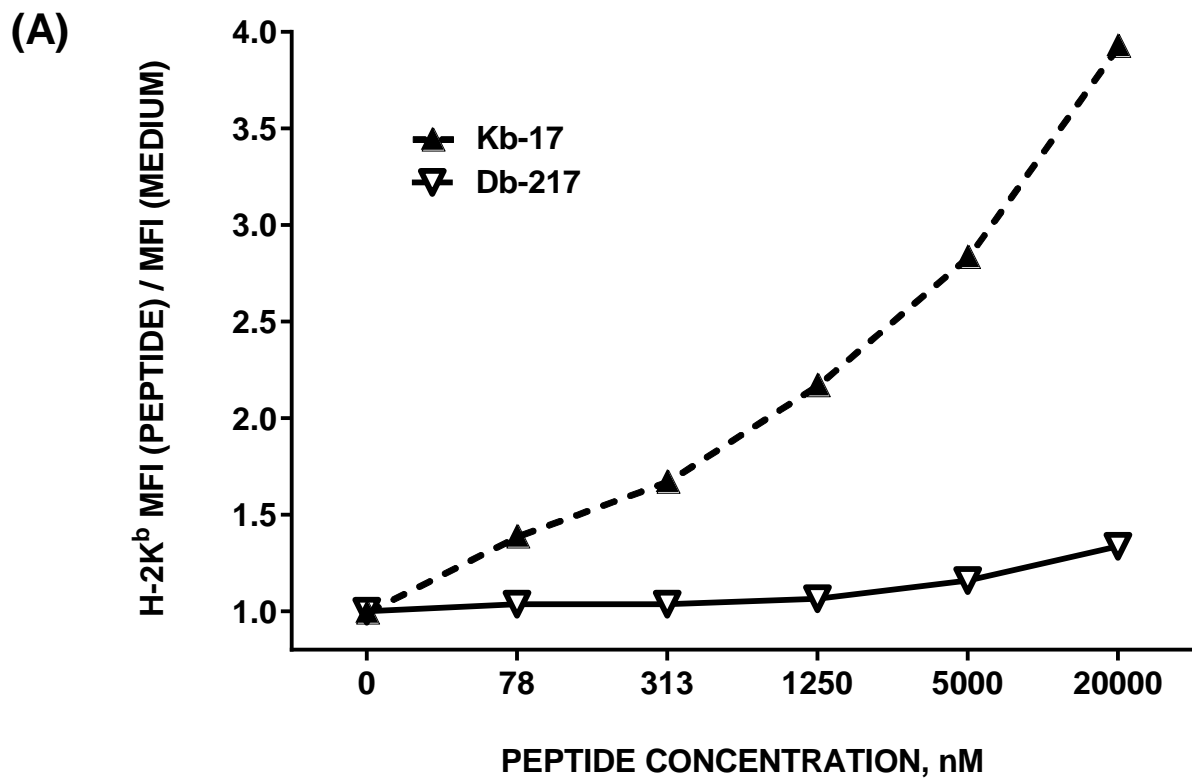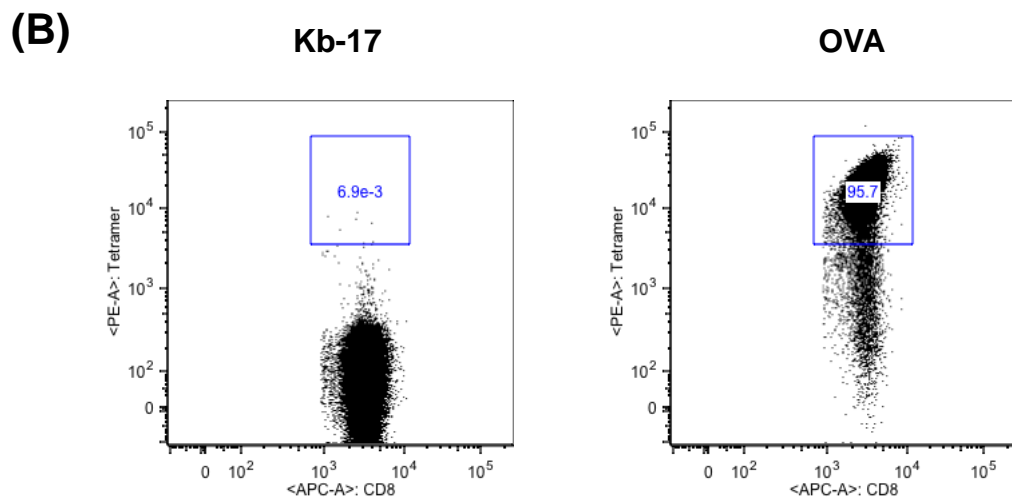

Figure S2

Supplement: Figure S2 — Gating strategy for detection of Kb-17-Tet+ cells and staining of splenocytes from transgenic mice with control caged MHC-tetramers. (A) Lymphocytes are first identified by forward (FSC) and side (SSC) scatter followed by dead cell exclusion based on Live/Dead Fixable Dead Cell Stain uptake; single cells are identified by FSC-A and FSC-H; and finally live single lymphocytes are analyzed for their CD3 and CD8 expression. (B) Splenocytes from transgenic OT-1 and P14 mice were stained with APC-labeled anti-CD8 mAbs and tetramers were generated using UV peptide exchange PE-labeled H-2Kb and H-2Db tetramers with OVA257-264 (SIINFEKL) and gp33 (KAVYNFATC) peptides, respectively. [file image_2.PDF]

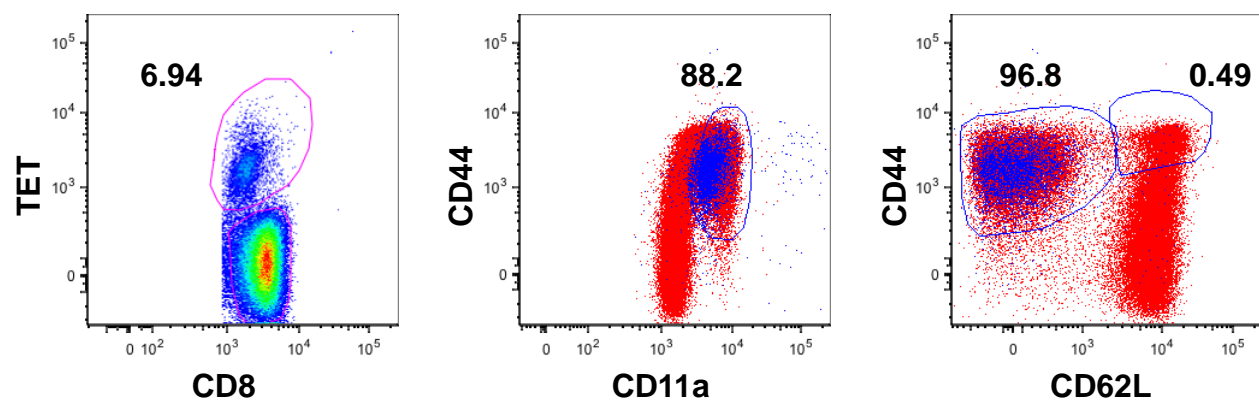

Figure S3

Supplement: Figure S3 — Immunization with Ad5-Kb-17 minigene vector expands Kb-17 specific CD8 T cells in the livers. Kb-17 tetramer positive liver CD8+ T cells (panel 1) harvested 7 days postboost have phenotype of antigen experienced CD11a+ (panel 2) CD44+CD62L− (panel 3) effector memory CD8 T cells (blue dots on the panels 2 and 3 represent tetramer-positive CD8+ T cells). The numbers represent percentage of tetramer-positive cells of total CD8 T cells (panel 1) and proportions of tetramer positive cells inside the corresponding gates among total tetramer-positive cells (panels 2 and 3). [file image_3.PDF]
