# Supplementary material for: Identification of a Novel CD8 T Cell Epitope Derived from Plasmodium berghei Protective Liver-Stage Antigen
Source: Front Immunol. 2018 Jan 29;9:91. doi: 10.3389/fimmu.2018.00091 (PMC5796907; doi:10.3389/fimmu.2018.00091)
Supplement: Supplementary file 4 [file table_1.PDF]

Table S1. Pb antigens and number of predicted CD8 T cell epitopes selected for the screening.

Gene	Product	epitope # (Kb)	epitope # (Db)	epitope # (total)
PBANKA_020970	zinc carboxy peptidase, putative	8	8	16
PBANKA_021130	cysteine desulfurase, putative (NFS)	2	6	8
PBANKA_021430	adapter-related protein, putative	6	15	21
PBANKA_031040	conserved Plasmodium protein, unknown function	5	4	9
PBANKA_040290	conserved Plasmodium protein, unknown function	15	24	39
PBANKA_040320	circumsporozoite (CS) protein (CSP)	0	1	1
PBANKA_051060	cell division cycle protein 20 homolog (CDC20)	5	6	11
PBANKA_071190	heat shock protein, putative (HSP70)	2	3	5
PBANKA_081890	heat shock protein 70, putative	0	1	1
PBANKA_083100	merozoite surface protein 1 (MSP1)	9	18	27
PBANKA_090210	sporozoite asparagine-rich protein (SLARP)	24	43	67
PBANKA_091440	heat shock protein hsp70 homologue, putative (UIS24)	0	1	1
PBANKA_092610	cupin-like protein, putative	1	6	7
PBANKA_093640	conserved Plasmodium protein, unknown function	3	5	8
PBANKA_100300	liver specific protein 2 (LISP2)	8	10	18
PBANKA_100320	conserved Plasmodium protein, unknown function	1	9	10
PBANKA_101040	conserved Plasmodium protein, unknown function	3	0	3
PBANKA_103100	conserved Plasmodium protein, unknown function	5	16	21
PBANKA_110580	TATA-box binding protein, putative,transcription initiation factor tfiid, putative (TBP)	2	0	2
PBANKA_112270	protein kinase, putative (TKL4)	2	3	5
PBANKA_112290	hexokinase, putative	14	2	16
PBANKA_121680	conserved Plasmodium protein, unknown function	8	19	27
PBANKA_123350	mRNA-binding protein PUF1 (PUF1)	8	21	29
PBANKA_131980	serine hydroxymethyltransferase, putative	5	17	22
PBANKA_133820	beta-hydroxyacyl-ACP dehydratase, putative (FabZ)	1	0	1

PBANKA_134010	L-lactate dehydrogenase (LDH)	2	3	5
PBANKA_140080	early transcribed membrane protein 13 (UIS3)	1	1	2
PBANKA_140700	conserved Plasmodium protein, unknown function	0	2	2
PBANKA_141800	conserved Plasmodium protein, unknown function	1	3	4
PBANKA_143230	cell traversal protein for ookinetes and sporozoites (CelTOS)	0	1	1
PBANKA_145490	conserved Plasmodium protein, unknown function (UIS8)	1	6	7

Table S2. Predicted H-2K^b-restricted epitopes from 31 selected Pb Ags.

Peptides			Score			Ranking			
Name	Gene	Sequence	RANKPEP	BIMAS	IEDB	RANKPEP	BIMAS	IEDB	Overall
Kb-1	PB001022.00.0	SVYFFFDKL	22.691	55	4.1	7	12	2	21
Kb-2	PB000547.01.0	NLYNNYVYL	22.037	100	98.1	11	3	44	58
Kb-3	PB001118.02.0	CYPYTYSYL	28.525	60	242.1	1	8	62	71
Kb-4	PB000687.02.0	IIIWNYPDL	18.792	26.4	29.7	31	27	18	76
Kb-5	PB001022.00.0	VNPIIYSQL	16.544	72	18.5	62	7	8	77
Kb-6	PB000547.01.0	DSYTNFNNL	20.59	72	148.3	19	6	53	78
Kb-7	PB000687.02.0	WNSHFFRNL	19.289	12	42.2	25	39	22	86
Kb-8	PB001118.02.0	KQFSIYSKL	18.814	40	81.3	30	18	39	87
Kb-9	PB001022.00.0	LAFSNYHTM	16.548	44	22.8	61	17	11	89
Kb-10	PB000993.00.0	KLYNEFNTL	18.126	60	121.6	34	9	49	92
Kb-11	PB000580.00.0	INSLNFEYL	20.89	13.2	94.2	16	36	43	95
Kb-12	PB000172.01.0	DGIKFFNKM	22.116	17.28	155.9	10	33	54	97
Kb-13	PB000547.01.0	KNYYNLQLL	25.616	7.2	104.6	2	53	45	100
Kb-14	PB001196.02.0	FVYVLFPVL	16.223	50	26.1	74	13	16	103
Kb-15	PB000547.01.0	AIYNCYKNL	15.413	158.4	32.9	96	1	20	117
Kb-16	PB000910.03.0	IGIFNYNKI	19.945	9.504	124.6	20	48	50	118
Kb-17	PB000950.01.0	IVSFSFQNM	16.816	13.2	70	50	38	34	122
Kb-18	PB000246.03.0	STYKNKSYL	20.851	0.66	20.6	17	96	10	123
Kb-19	PB000580.00.0	NVQKNYNQL	19.879	86.4	1718.3	21	4	98	123
Kb-20	PB000580.00.0	CIIIDFNKL	21.547	14.4	554.6	14	34	78	126
Kb-21	PB000950.01.0	SEYDKYQDF	18.966	4.752	75.8	29	61	37	127
Kb-22	PB000246.03.0	STHTEYTNL	17.265	26.4	195.3	41	29	57	127
Kb-23	PB001118.02.0	LAITNYDKL	16.802	26.4	133.6	51	30	52	133
Kb-24	PB000547.01.0	IPIKNYYNL	17.277	26.4	313.2	40	28	66	134

Kb-25	PB000993.00.0	KLFFYYKLL	8.531	48	14.7	116	15	6	137
Kb-26	PB000307.01.0	TNYCCPTYM	19.277	6	213.8	27	55	58	140
Kb-27	PB000172.01.0	TMYSLYESI	10.847	30	17.9	111	24	7	142
Kb-28	PB000252.03.0	IYSPHFAHL	18.731	11	332	32	43	67	142
Kb-29	PB000172.01.0	KYVPIFEDL	22.394	10	1183.9	8	45	89	142
Kb-30	PB000580.00.0	NQITNYKKM	22.231	28.8	3790	9	26	112	147
Kb-31	PB000547.01.0	TIFNVYWHL	9.037	48	48.4	115	14	24	153
Kb-32	PB000579.01.0	NLYNTYKYL	15.921	120	398.6	81	2	72	155
Kb-33	PB000660.02.0	DQSDKYTKL	22.826	24	4833.6	6	32	120	158
Kb-34	PB001022.00.0	NQYLFYLQI	12.204	30	53.4	110	23	28	161
Kb-35	PB000838.03.0	KSDDGYQKM	21.77	5.76	1250.3	13	59	90	162
Kb-36	PB000185.00.0	LKYYISQKL	16.508	2.178	73.7	63	69	36	168
Kb-37	PB000702.00.0	KMHFFFNYV	15.998	1.2	19.2	77	83	9	169
Kb-38	PB000789.01.0	CSLYLSDKL	17.507	1.1	120.9	36	85	48	169
Kb-39	PB000993.00.0	IDSDNFEKL	19.329	13.2	3426.3	24	37	108	169
Kb-40	PB000993.00.0	SYIFMLELL	16.779	1.1	70.7	53	86	35	174
Kb-41	PB000579.01.0	NNINIYSDI	17.443	7.2	711.9	37	54	83	174
Kb-42	PB000580.00.0	DNDDNYENM	21.773	5.76	3514.9	12	58	109	179
Kb-43	PB000687.02.0	SNLDLYRNI	15.55	9.504	92.1	92	50	42	184
Kb-44	PB000172.01.0	NAFEQYKEL	7.58	57.6	174.4	118	11	56	185
Kb-45	PB001022.00.0	YYLHMLNKL	19.686	1.2	731	23	82	84	189
Kb-46	PB001022.00.0	KHHYNYTYS	19.14	2	1315.4	28	70	91	189
Kb-47	PB000307.01.0	SSYKKYNNK	16.343	1.584	115	70	74	46	190
Kb-48	PB001022.00.0	YIYLQYITI	7.398	36	127	119	20	51	190
Kb-49	PB001188.02.0	SYIDIYNDI	15.957	9.504	261.6	79	49	63	191
Kb-50	PB000579.01.0	NNNDHYQMM	13.671	34.56	287.5	109	21	64	194
Kb-51	PB000727.00.0	YIMGEFNDL	16.792	14.4	3299	52	35	107	194
Kb-52	PB000042.03.0	NIPNNFENI	19.727	10.8	8289.6	22	44	128	194
Kb-53	PB000993.00.0	CSYIFMLEL	10.64	5	37.5	114	60	21	195
Kb-54	PB001188.02.0	CFTTKFTDL	16.561	12	1660.1	60	40	96	196

Kb-55	PB000185.00.0	SGSNTYDDL	16.398	26.4	1700.8	68	31	97	196
Kb-56	PB001022.00.0	YMSYIFNDI	17.093	3.6	1178.2	47	63	88	198
Kb-57	PB000579.01.0	SNINLYNTY	15.954	0.95	57.7	80	89	30	199
Kb-58	PB000950.01.0	CVYPNFNMK	25.017	0.6	1776.4	3	98	99	200
Kb-59	PB000579.01.0	NNLYNFNNY	23.118	0.432	1499.9	5	104	93	202
Kb-60	PB000580.00.0	GNKYNYKYI	17.44	1.728	1647.8	38	72	95	205
Kb-61	PB001022.00.0	YNYTYSVNL	4.856	7.92	59.1	124	52	32	208
Kb-62	PB000579.01.0	YNFNNYKNY	16.566	1.728	511.3	59	73	77	209
Kb-63	PB000834.02.0	VFQDIYNVL	4.735	86.4	571.3	125	5	80	210
Kb-64	PB000910.03.0	YFYLSYILI	2.68	30	224.3	129	25	59	213
Kb-65	PB000307.01.0	CPPNGVNTM	19.287	3.6	8017.5	26	62	125	213
Kb-66	PB000993.00.0	LKFYMYSQF	15.523	0.396	23.9	95	106	13	214
Kb-67	PB000172.01.0	KKKIIYQAM	17.157	1.44	1341.5	46	76	92	214
Kb-68	PB001022.00.0	LPYDTYKNI	10.781	47.52	888.6	113	16	87	216
Kb-69	PB000702.00.0	NTSGNYPKF	24.588	0.6	3825.1	4	99	113	216
Kb-70	PB000172.01.0	CLYGNTKQL	15.298	12	465.6	101	42	74	217
Kb-71	PB000838.03.0	CNYYNNGEI	15.34	1.8	120	100	71	47	218
Kb-72	PB000579.01.0	DNIINLQNL	17.035	1.44	1562.2	48	77	94	219
Kb-73	PB000172.01.0	CSSSSFMGL	15.298	10	397.8	102	47	71	220
Kb-74	PB000307.01.0	GNYVIQRAL	15.626	6	507.1	89	56	76	221
Kb-75	PB000687.02.0	KNILNSNYL	15.729	1.584	238.9	87	75	61	223
Kb-76	PB000789.01.0	KNVNIFTKI	15.986	3.6	591.3	78	65	81	224
Kb-77	PB000579.01.0	KNKNGFNYL	16.287	2.88	734.2	72	67	85	224
Kb-78	PB000307.01.0	IIPCKYTNY	15.904	2.376	484.9	82	68	75	225
Kb-79	PB000579.01.0	YNVPQFDNL	15.375	12	842.6	99	41	86	226
Kb-80	PB000950.01.0	SSSYFYSFK	17.162	0.22	304	45	119	65	229
Kb-81	PB001022.00.0	INHNFYLTF	10.802	0.792	57.5	112	91	29	232
Kb-82	PB000727.00.0	FVYELKKGL	3.859	0.72	24.5	127	94	15	236
Kb-83	PB000727.00.0	LVMDWYSAN	6.697	0.264	4.4	120	114	3	237
Kb-84	PB000172.01.0	GLQNIYAEL	5.496	60	2669.8	123	10	105	238

Kb-85	PB001022.00.0	NNNTLYKIL	6.586	34.56	1970.8	121	22	101	244
Kb-86	PB000910.03.0	CNPKNSSHY	20.938	0.143	3114.7	15	124	106	245
Kb-87	PB000727.00.0	VVNDAVGTL	2.828	1.2	82.7	128	84	40	252
Kb-88	PB001118.02.0	TYSYLMEYL	15.815	1	562.9	85	88	79	252
Kb-89	PB000727.00.0	GNFDKDLPL	-8.095	0.288	7.6	139	111	4	254
Kb-90	PB000727.00.0	CAYQKGKSA	-1.701	0.6	32.6	135	100	19	254
Kb-91	PB000580.00.0	DSNNNFDKM	15.538	10	4109.1	93	46	115	254
Kb-92	PB000579.01.0	KNIYNHPNY	16.414	0.036	168.7	66	135	55	256
Kb-93	PB001022.00.0	ICIYKNQYL	15.023	1.32	333.3	107	81	69	257
Kb-94	PB000727.00.0	ISYGTLEEF	2.654	0.165	14.1	130	123	5	258
Kb-95	PB000246.03.0	CYIIFYRKG	16.402	0.2	387	67	121	70	258
Kb-96	PB000817.02.0	YCYGVKSSL	16.622	0.5	1961.8	56	102	100	258
Kb-97	PB000817.02.0	AGGGMPGGM	16.727	1.32	8052.5	54	79	126	259
Kb-98	PB000838.03.0	GNYYFINPF	7.675	0.216	46	117	120	23	260
Kb-99	PB000727.00.0	SNGCYYEPD	2.492	0.264	24.2	132	115	14	261
Kb-100	PB000246.03.0	KNFVNYQSK	16.596	0.576	2148.7	58	101	102	261
Kb-101	PB001022.00.0	CYFYFLSKK	16.656	0.02	332.4	55	139	68	262
Kb-102	PB001188.02.0	RICESYKAL	4.535	38.016	4579.6	126	19	118	263
Kb-103	PB000727.00.0	ILIDWTKGF	-4.2	0.095	4	136	127	1	264
Kb-104	PB000246.03.0	NNPYNFNYE	17.638	0.432	7673.3	35	105	124	264
Kb-105	PB000809.02.0	NILKKYRSF	15.532	0.864	592.3	94	90	82	266
Kb-106	PB001118.02.0	RDLNNYQDI	15.639	7.92	8159.6	88	51	127	266
Kb-107	PB000579.01.0	NRNNNFNMM	16.254	3.6	10560.6	73	64	131	268
Kb-108	PB000660.02.0	KQINEFSKY	17.364	0.3	5094.9	39	109	121	269
Kb-109	PB000579.01.0	TNYLTRNSL	0.506	0.72	90.6	134	95	41	270
Kb-110	PB001118.02.0	SAVDNFGNT	15.769	1.32	2571.1	86	80	104	270
Kb-111	PB001188.02.0	FRYIKFSHF	15.002	0.45	228.2	108	103	60	271
Kb-112	PB000687.02.0	NNDNNLPLL	17.263	0.24	4072.4	42	116	114	272
Kb-113	PB000727.00.0	TNFRAVRAS	-7.346	0.288	50.7	138	110	27	275
Kb-114	PB001118.02.0	CIDNIFSLY	17.257	0.072	2394.5	43	130	103	276

Kb-115	PB000838.03.0	FPFPNFEMI	15.05	6	4151.6	106	57	116	279
Kb-116	PB001022.00.0	VYFFFDKLM	2.575	0.24	58.4	131	118	31	280
Kb-117	PB000579.01.0	PCPPKPSNL	18.441	0.3	23521	33	108	140	281
Kb-118	PB000727.00.0	DNNEKSVGF	-5.974	0.048	23.4	137	133	12	282
Kb-119	PB000727.00.0	VGFTFSFPC	-13.954	0.317	66.8	142	107	33	282
Kb-120	PB000579.01.0	NVDNNYNMI	15.873	1.44	6149.8	83	78	122	283
Kb-121	PB000579.01.0	NRGNNFEDF	20.813	0.09	15785.8	18	128	139	285
Kb-122	PB000727.00.0	VNVLQSASS	-11.683	0.132	48.7	140	125	25	290
Kb-123	PB000950.01.0	VSFSFQNMF	0.752	0.072	49.6	133	131	26	290
Kb-124	PB000727.00.0	VNQLRISYG	-12.791	0.036	26.7	141	136	17	294
Kb-125	PB000838.03.0	SCYCNYYNN	15.584	1.1	4503.8	90	87	117	294
Kb-126	PB001188.02.0	KDFKNFEKY	15.849	0.72	4720.6	84	93	119	296
Kb-127	PB000579.01.0	NRSNVFNEL	15.377	3.6	11165.1	98	66	132	296
Kb-128	PB000660.02.0	ISYEKKNKF	5.784	0.024	75.8	122	137	38	297
Kb-129	PB000838.03.0	QCGPPYKYY	16.352	0.72	15171.9	69	92	136	297
Kb-130	PB000307.01.0	NPINNYQQN	16.898	0.24	15775	49	117	138	304
Kb-131	PB000547.01.0	NCYKNLMNF	16.003	0.18	3547.1	76	122	110	308
Kb-132	PB000172.01.0	SGYVNFFNK	15.296	0.66	3741.3	103	97	111	311
Kb-133	PB000307.01.0	SCSENYPNN	16.326	0.264	9576.9	71	112	130	313
Kb-134	PB001036.02.0	CTKVFKEKL	15.262	0.02	444.9	104	140	73	317
Kb-135	PB000892.03.0	NKDFNFPFF	17.225	0.018	12061.8	44	141	133	318
Kb-136	PB000838.03.0	PGSFPFPNF	16.605	0.036	8599.6	57	134	129	320
Kb-137	PB000579.01.0	PRSKNYPVF	16.502	0.022	12386.1	64	138	134	336
Kb-138	PB000838.03.0	NSENNSQDM	15.571	0.264	12401.4	91	113	135	339
Kb-139	PB001118.02.0	KCSVDFSDK	16.141	0.1	29250.5	75	126	142	343
Kb-140	PB000307.01.0	YKSGNSSKY	16.451	0.01	24411.3	65	142	141	348
Kb-141	PB000834.02.0	SAEYNPSKI	15.398	0.066	6874.9	97	132	123	352
Kb-142	PB000580.00.0	LRDYNFNDT	15.1	0.079	15207.9	105	129	137	371

Table S3. Predicted H-2D^b-restricted epitopes from 31 selected Pb Ags.

Peptides			Score				Ranking				
Name	Gene	Sequence	RANKPEP	BIMAS	SYFPEITHI	IEDB	RANKPEP	BIMAS	SYFPEITHI	IEDB	Overall
Db-1	PBANKA_123350	NMIRNIDNI	26.524	11889.854	27	4.1	7	1	8	7	23
Db-2	PBANKA_020970	LAITNYDKL	25.223	1057.831	28	3.6	11	31	6	5	53
Db-3	PBANKA_123350	SNISNDDYL	24.834	1019.818	29	8.2	13	33	3	11	60
Db-4	PBANKA_040290	KQVLNLDTL	20.55	1679.2	30	4	39	20	2	6	67
Db-5	PBANKA_121680	KAIQNSDEI	20.283	1655.549	31	2.2	46	23	1	1	71
Db-6	PBANKA_121680	TVIKNEDEI	20.505	7794.194	27	21.5	41	2	9	21	73
Db-7	PBANKA_090210	NSINNSNTI	23.607	3281.04	26	57.3	19	12	23	30	84
Db-8	PBANKA_091440	NAIINISAI	20.636	2116.8	26	10.5	38	16	24	14	92
Db-9	PBANKA_040290	SQLKNIFQI	19.638	3967.73	26	43	51	7	20	27	105
Db-10	PBANKA_040290	SLISNIKYL	20.297	1580.705	27	63.6	45	24	13	31	113
Db-11	PBANKA_083100	NALKNNDML	17.192	7166.88	27	8.2	107	4	11	12	134
Db-12	PBANKA_140700	GVHVNADLI	26.19	1673.666	25	1579.1	8	21	36	72	137
Db-13	PBANKA_021130	FGNMNISFL	20.875	302.4	26	16.2	34	74	30	17	155
Db-14	PBANKA_131980	STYKNKSYL	18.258	755.758	26	12.4	79	39	26	15	159
Db-15	PBANKA_123350	IAVENCNNI	18.696	1524.096	27	757.3	68	25	14	55	162
Db-16	PBANKA_121680	NQITNYKKM	25.096	314.388	24	100.1	12	73	66	36	187
Db-17	PBANKA_020970	NSIKNIEDI	16.206	7776.065	27	285.6	130	3	10	46	189
Db-18	PBANKA_051060	RNLNNLNYI	23.82	232.848	25	437	17	82	43	50	192
Db-19	PBANKA_100320	YMIRNFSLF	23.761	51.192	26	10.4	18	131	33	13	195
Db-20	PBANKA_083100	YVIRNPYQL	18.661	3631.824	22	26.6	69	8	106	22	205
Db-21	PBANKA_040290	STPTNNSTI	17.294	248.371	29	12.7	105	81	5	16	207
Db-22	PBANKA_131980	STNSNLTFL	16.207	354.816	28	3.3	129	68	7	4	208
Db-23	PBANKA_131980	QSLQNYNSL	14.43	4687.2	26	19	168	6	19	18	211
Db-24	PBANKA_040290	LAFSNYHTM	17.014	432	26	4.3	114	62	29	8	213
Db-25	PBANKA_083100	AKMLNMDSM	20.351	194.53	25	95.5	44	93	44	34	215
Db-26	PBANKA_123350	YSNYNDPNM	24.558	455.7	22	100.3	15	55	112	37	219

Db-27	PBANKA_100320	STFLNFSTI	17.332	248.371	26	5.7	102	80	31	9	222
Db-28	PBANKA_021430	SSKNNVSFI	16.342	1212.672	25	70.2	126	29	38	32	225
Db-29	PBANKA_051060	WNINNGECI	27.22	551.85	24	8486.1	4	47	56	121	228
Db-30	PBANKA_140700	YHIINNESI	17.353	802.691	25	1034.6	99	37	39	62	237
Db-31	PBANKA_090210	NLYNNYVYL	27.04	166.32	23	197.8	6	104	88	43	241
Db-32	PBANKA_021430	NQNTNLINI	18.134	417.48	24	99.3	82	63	63	35	243
Db-33	PBANKA_123350	FINNNMTPI	31.609	58.8	23	39.5	2	127	92	25	246
Db-34	PBANKA_090210	INLQNLNYI	17.158	167.651	27	55	109	101	17	29	256
Db-35	PBANKA_090210	SSNSNTILL	12.913	3437.28	26	5.9	218	9	21	10	258
Db-36	PBANKA_121680	KALQNQIEL	11.718	997.92	29	2.7	222	35	4	2	263
Db-37	PBANKA_040290	NVMKNVNTI	17.337	2542.277	22	166.5	101	15	107	42	265
Db-38	PBANKA_051060	GSNDNSIYL	16.76	1302	25	2345.3	121	27	37	80	265
Db-39	PBANKA_123350	KQMNNNSSM	16.899	506.088	24	203	118	51	58	44	271
Db-40	PBANKA_131980	NATLNLEHL	13.244	1990.8	27	334.5	208	17	12	48	285
Db-41	PBANKA_121680	SSNSNSNSL	12.14	3437.28	26	70.8	221	10	22	33	286
Db-42	PBANKA_083100	NSTINKDAL	14.534	6572.626	23	106.1	166	5	80	38	289
Db-43	PBANKA_021130	YIIFNGCQI	23.103	211.68	21	137.3	20	90	145	40	295
Db-44	PBANKA_021130	TQNDNSDKL	17.47	764.969	25	9609.9	95	38	40	124	297
Db-45	PBANKA_090210	KQNINRYNI	18.808	293.447	22	127.6	65	77	117	39	298
Db-46	PBANKA_121680	DSNNNFDKM	29.714	13.555	25	2461.1	3	162	51	83	299
Db-47	PBANKA_090210	SNLRNSNLI	14.12	614.719	26	386.7	180	45	27	49	301
Db-48	PBANKA_040290	MCVSNFIQL	18.294	435.456	24	5738.4	78	60	61	104	303
Db-49	PBANKA_090210	SMTSNDSSI	9.179	1802.709	26	26.8	238	18	25	23	304
Db-50	PBANKA_051060	YSRENEENI	14.9	533.438	25	619	159	49	42	54	304
Db-51	PBANKA_143230	FCFFNVLCL	24.333	168	22	1092.7	16	100	122	66	304
Db-52	PBANKA_021430	RPPPNNDTI	21.177	29.265	26	3926.6	29	144	34	97	304
Db-53	PBANKA_021130	VQKMNIDLL	15.589	512.919	24	904.6	145	50	57	59	311
Db-54	PBANKA_123350	NSLKNSIII	10.308	3281.04	24	21.4	229	13	53	20	315
Db-55	PBANKA_040290	LHIKNKQVL	17.071	742.017	24	6320.5	111	40	55	111	317
Db-56	PBANKA_090210	NVIANDPLI	17.31	1245.716	22	2336.6	104	28	108	79	319

Db-57	PBANKA_103100	YILKNNIYL	15.146	302.4	24	42.2	157	75	67	26	325
Db-58	PBANKA_123350	SMGANFVNV	16.87	187.704	23	49.3	119	96	86	28	329
Db-59	PBANKA_134010	GAHGNKMVL	17.992	1789.2	23	14516.1	84	19	82	148	333
Db-60	PBANKA_121680	RNLTNEQNL	19.102	162.994	23	2650	59	105	89	85	338
Db-61	PBANKA_112270	NCLNNENVI	24.605	207.446	22	7284.4	14	91	119	115	339
Db-62	PBANKA_145490	NIPNNFENI	25.83	139.356	25	21843.4	9	110	48	177	344
Db-63	PBANKA_021130	NGNNNKEII	18.485	1068.582	23	17047.6	75	30	84	156	345
Db-64	PBANKA_103100	NSVDNIIII	9.647	3281.04	24	829	231	14	54	56	355
Db-65	PBANKA_021430	YKNRNSDKI	22.251	10.856	23	1071.1	23	169	98	65	355
Db-66	PBANKA_090210	KNYYNLQLL	19.932	30.492	25	8834.1	47	141	50	122	360
Db-67	PBANKA_051060	ASGSNDNSI	11.479	446.586	27	1290.4	223	56	16	69	364
Db-68	PBANKA_100300	FPFPNFEMI	18.998	29.265	24	2682.4	60	145	73	87	365
Db-69	PBANKA_123350	RNIDNINTI	18.371	232.848	24	14165.9	77	83	68	146	374
Db-70	PBANKA_020970	ICIDNIFSL	18.242	435.456	24	21583.4	80	61	62	176	379
Db-71	PBANKA_090210	RTFCNYNNI	19.793	94.08	19	29.9	49	119	188	24	380
Db-72	PBANKA_103100	SKFKNRVFL	14.158	179.493	25	876.4	179	98	45	58	380
Db-73	PBANKA_090210	YIKNNFDIL	22.96	100.336	20	2123	21	116	167	76	380
Db-74	PBANKA_103100	YPNFNMKYI	22.368	12.348	22	1029.3	22	167	132	61	382
Db-75	PBANKA_123350	TNVENNENM	13.378	1486.616	27	13104.2	201	26	15	144	386
Db-76	PBANKA_112290	FGGTNFRAV	19.516	10.8	23	1159.1	52	170	99	68	389
Db-77	PBANKA_090210	NNIENNENI	15.361	551.85	26	18061.1	153	48	28	160	389
Db-78	PBANKA_083100	MCANNYCEI	27.144	84.672	22	12413.6	5	121	126	138	390
Db-79	PBANKA_083100	AIQNNMPTM	21.045	60	23	12490.9	31	126	91	142	390
Db-80	PBANKA_040290	YANQNVYPV	18.471	30	20	2.8	76	142	170	3	391
Db-81	PBANKA_140080	NSLKNIDTA	17.559	396.738	19	491.2	93	65	184	52	394
Db-82	PBANKA_021430	FLRKNYYNL	18.714	83.825	22	2333.6	67	122	127	78	394
Db-83	PBANKA_090210	NNNVNSEVL	16.86	218.988	24	7840.4	120	86	70	118	394
Db-84	PBANKA_090210	SNVDNNYNM	13.721	627.264	25	7799.4	193	44	41	117	395
Db-85	PBANKA_020970	TCGFNCPIL	30261.5	443.52	21	30261.5	1	57	143	199	400
Db-86	PBANKA_123350	MFLNNENYI	22.139	8.962	23	5947.2	25	173	100	106	404

Db-87	PBANKA_100300	ANDRNTSYM	17.668	3.894	26	3043.4	90	188	35	92	405
Db-88	PBANKA_112270	MTNANICSL	20.41	96.768	21	3625.5	43	118	150	96	407
Db-89	PBANKA_121680	NDIANKDNI	19.105	29.92	24	11906.6	58	143	72	136	409
Db-90	PBANKA_131980	CEQFNPDLI	19.385	14.348	23	6061	54	158	94	107	413
Db-91	PBANKA_121680	ISATNDCII	13.943	321.542	22	205.3	184	72	115	45	416
Db-92	PBANKA_121680	INSLNFEYL	14.297	157.671	25	4766.9	172	106	46	100	424
Db-93	PBANKA_090210	NNVSNTTSI	9.475	232.848	26	1659.8	235	85	32	74	426
Db-94	PBANKA_103100	NEITNINLI	17.954	8.256	24	2691.3	85	178	76	88	427
Db-95	PBANKA_103100	ECVHNLSVI	21.855	12.701	24	18138.5	27	164	75	162	428
Db-96	PBANKA_090210	DSYTNFNNL	16.089	39.06	24	2651.4	135	137	71	86	429
Db-97	PBANKA_040290	SAKNNKKKL	13.896	488.031	24	11531.6	188	53	60	133	434
Db-98	PBANKA_141800	NVKTNSDNI	15.149	843.527	21	6215.6	156	36	141	109	442
Db-99	PBANKA_040290	FQNNNLRNF	25.243	4.26	23	16049.5	10	185	102	152	449
Db-100	PBANKA_141800	NNIENKKNI	15.259	495.966	24	24840.8	154	52	59	184	449
Db-101	PBANKA_031040	FLFFNTPQI	15.557	116.424	22	1149.5	147	113	124	67	451
Db-102	PBANKA_071190	WRNENVDII	18.485	12.681	23	7099	74	166	97	114	451
Db-103	PBANKA_123350	HQMKNDNNL	12.999	1052.05	22	3335.5	217	32	109	94	452
Db-104	PBANKA_021130	LMSLNDIAL	5.804	702.354	22	445.6	250	42	110	51	453
Db-105	PBANKA_090210	KLTCNFKFL	15.744	54.886	22	571.6	143	129	128	53	453
Db-106	PBANKA_040290	ISAINTLIM	9.611	669.6	21	164	233	43	142	41	459
Db-107	PBANKA_121680	SNSLNKKYI	11.185	363.709	24	5219.1	224	67	65	103	459
Db-108	PBANKA_040290	TEYWNDQFI	20.916	2.966	23	10610.4	33	196	104	127	460
Db-109	PBANKA_121680	EQINNEMEI	16.378	22.093	24	6623.3	125	152	74	112	463
Db-110	PBANKA_040290	GVNVNNEDI	12.67	1673.666	23	15721.7	219	22	83	151	475
Db-111	PBANKA_123350	VDIYNAESI	18.135	10.114	27	32019.9	81	171	18	206	476
Db-112	PBANKA_040290	NTSPNLYNI	17.351	94.08	22	11534.9	100	120	125	134	479
Db-113	PBANKA_090210	NNNNNNSNM	21.149	66	21	23111.9	30	124	151	180	485
Db-114	PBANKA_083100	CLYGNTKQL	18.997	439.085	20	31840.6	61	58	164	204	487
Db-115	PBANKA_123350	RLNNNINII	20.701	116.424	18	12964.5	37	112	196	143	488
Db-116	PBANKA_100300	FNTVNNEDI	8.791	153.292	25	3194.9	242	107	47	93	489

Db-117	PBANKA_090210	TNQSNNDNM	9.625	412.949	24	10969.7	232	64	64	129	489
Db-118	PBANKA_051060	IIIWNYPDL	15.957	217.728	21	9502	139	88	144	123	494
Db-119	PBANKA_100300	NLFRNVKPI	14.552	116.424	21	1472.4	165	114	148	71	498
Db-120	PBANKA_103100	VILKNEKNI	16.188	74.681	23	16754.6	131	123	90	154	498
Db-121	PBANKA_083100	TLVPNKYGL	9.275	3366.902	23	20191.2	237	11	81	169	498
Db-122	PBANKA_092610	SRIHNNGLI	14.847	50.854	25	17666.3	161	132	49	157	499
Db-123	PBANKA_090210	IPIKNYYNL	13.136	45.723	23	1456.2	210	134	93	70	507
Db-124	PBANKA_090210	KSINNISTT	17.878	55.242	18	4729	87	128	197	99	511
Db-125	PBANKA_040290	KIENNYFYI	20.724	1.145	21	4850.7	36	220	160	102	518
Db-126	PBANKA_092610	QKYINSNYI	15.479	22.344	22	2789.8	150	151	130	89	520
Db-127	PBANKA_123350	INDNNSTHM	19.64	2.804	23	20000.9	50	199	105	168	522
Db-128	PBANKA_040290	KENNNYHVI	19.33	0.757	21	2394.7	55	228	161	81	525
Db-129	PBANKA_131980	FLYLNKKYF	17.248	2.53	22	2590	106	201	135	84	526
Db-130	PBANKA_092610	NYYLNTQYI	13.311	6.821	23	297.6	203	180	101	47	531
Db-131	PBANKA_040290	NSMENVIAV	7.715	167.4	20	20.7	245	103	166	19	533
Db-132	PBANKA_112290	SLSSNMKQL	13.308	439.085	23	25869.1	204	59	85	186	534
Db-133	PBANKA_090210	VLTNNLFKI	17.359	17.184	22	16334.1	98	154	131	153	536
Db-134	PBANKA_134010	NNCPNAFII	20.528	64.68	20	31263.8	40	125	168	203	536
Db-135	PBANKA_121680	DCIINSKHI	19.167	12.701	23	35802.1	57	165	96	218	536
Db-136	PBANKA_131980	NKNINILFL	15.622	31.92	21	4828.9	144	140	154	101	539
Db-137	PBANKA_100320	NILINDINI	13.325	103.723	21	1910.5	202	115	149	75	541
Db-138	PBANKA_040290	FQINNNTLY	18.525	15.336	18	6270.3	73	157	201	110	541
Db-139	PBANKA_100320	ISHLNNRKL	6.433	192.175	22	2458.9	247	94	120	82	543
Db-140	PBANKA_131980	LYIKNNDKI	13.473	8.59	25	7313.5	199	176	52	116	543
Db-141	PBANKA_021430	ISRYNIIIL	5.543	472.47	20	2265.2	251	54	163	77	545
Db-142	PBANKA_123350	INIINSNII	10.994	167.651	21	1584.8	225	102	147	73	547
Db-143	PBANKA_090210	NNIENSNDI	10.392	232.848	24	19923.4	228	84	69	166	547
Db-144	PBANKA_121680	YLKKNEFNL	16.413	41.074	20	8421.5	123	136	169	120	548
Db-145	PBANKA_090210	LHILNNNNL	8.927	348.365	22	11103.9	240	69	113	131	553
Db-146	PBANKA_020970	LCMSNKSKL	9.808	190.142	22	6215.2	230	95	121	108	554

Db-147	PBANKA_090210	IMDPNENII	14.813	29.007	21	3016.1	163	146	155	91	555
Db-148	PBANKA_121680	GNKYNYKYI	19.412	32.599	21	32891.9	53	139	153	211	556
Db-149	PBANKA_083100	KQIVNLKYA	15.527	25.304	20	3409.6	148	149	172	95	564
Db-150	PBANKA_131980	SEFINQSIL	13.486	8.649	22	1027.1	198	175	133	60	566
Db-151	PBANKA_021430	TSLELLDMI	3.947	205.288	20	867	254	92	165	57	568
Db-152	PBANKA_121680	KEIKNILNI	13.936	2.724	24	5870.1	185	200	78	105	568
Db-153	PBANKA_040290	VNVNNEDIF	19.929	1.987	22	23279.2	48	204	136	182	570
Db-154	PBANKA_090210	NRNNNFNMM	21.235	5.46	20	24966.7	28	183	174	185	570
Db-155	PBANKA_021430	SEINNLKEV	14.943	1.112	24	6995.4	158	221	79	113	571
Db-156	PBANKA_090210	NMLNNAYNT	15.753	255.96	15	12415.7	142	79	214	139	574
Db-157	PBANKA_083100	IDGNNTEFI	16.391	2.809	24	21413.8	124	198	77	175	574
Db-158	PBANKA_083100	FSYSCHKYL	19.178	0.716	17	2995.5	56	229	209	90	584
Db-159	PBANKA_090210	NVDNNYNMI	17.536	41.665	18	19185.8	94	135	198	163	590
Db-160	PBANKA_100300	NSENNSQDM	13.907	54.87	22	14324.3	187	130	129	147	593
Db-161	PBANKA_031040	FQVCVDVYM	15.394	7.515	18	1062.7	151	179	202	64	596
Db-162	PBANKA_090210	IIHNNLKKI	18.619	8.679	21	28768.6	71	174	158	193	596
Db-163	PBANKA_040290	LVSSNEEDI	5.285	590.469	22	26644.3	252	46	111	188	597
Db-164	PBANKA_083100	YKKNNNTCI	16.105	11.261	21	12417.5	134	168	157	140	599
Db-165	PBANKA_040290	CIYKNQYLF	22.177	1.584	19	20640.9	24	212	193	172	601
Db-166	PBANKA_121680	NVQKNYNQL	10.479	1008.84	20	23073	227	34	162	178	601
Db-167	PBANKA_090210	PNNNNTSAM	18.957	6.6	20	25875.6	62	181	173	187	603
Db-168	PBANKA_145490	KSKKNSKIL	6.091	216.549	22	14632.6	248	89	118	149	604
Db-169	PBANKA_100300	NYYNNGEII	16.037	16.165	21	18030.2	137	156	156	159	608
Db-170	PBANKA_083100	TIKFNLDGI	9.4	185.422	23	27057.9	236	97	87	189	609
Db-171	PBANKA_090210	NTNNNNLKL	14.837	27.552	20	11155.9	162	148	171	132	613
Db-172	PBANKA_093640	LNNKNYKQI	16.221	46.57	21	30660.1	128	133	152	200	613
Db-173	PBANKA_123350	TNYCCPTYM	17.787	1.742	15	4623.2	89	207	220	98	614
Db-174	PBANKA_090210	HNINNNNDL	9.481	332.64	22	29145	234	71	114	195	614
Db-175	PBANKA_103100	FSFQNMFKK	20.433	0.953	15	10905.3	42	223	222	128	615
Db-176	PBANKA_090210	NINNNNDLF	21.994	1.422	20	32186.6	26	214	180	208	628

Db-177	PBANKA_131980	NSLKNVDED	5.818	396.738	19	11016.8	249	66	185	130	630
Db-178	PBANKA_071190	NSLENYCYG	13.117	16.74	18	1046	214	155	200	63	632
Db-179	PBANKA_021430	GLKVNKEII	8.254	296.211	22	29490.8	244	76	116	196	632
Db-180	PBANKA_090210	NECINFNSL	17.32	3.276	22	32764.1	103	192	134	210	639
Db-181	PBANKA_090210	NVKNNETYF	18.098	1.78	20	21350.4	83	206	178	173	640
Db-182	PBANKA_141800	NIYDNKLFL	8.9	178.92	21	16870.5	241	99	146	155	641
Db-183	PBANKA_103100	LIVKNSLDI	4.747	152.41	22	17956.4	253	108	123	158	642
Db-184	PBANKA_123350	PNNINENHI	17.845	3.169	20	30157.7	88	193	176	198	655
Db-185	PBANKA_103100	YFKNNINLM	16.054	3.629	19	12038.5	136	191	192	137	656
Db-186	PBANKA_100320	SLLYNVDIA	13.286	133.795	15	9628.3	206	111	215	125	657
Db-187	PBANKA_090210	DNNYNMINM	18.85	1.98	20	34320	64	205	177	216	662
Db-188	PBANKA_131980	PYNFNYEFI	13.618	1.617	22	10174.3	194	211	138	126	669
Db-189	PBANKA_083100	HVCINTRDI	6.873	706.188	19	31866.1	246	41	183	205	675
Db-190	PBANKA_092610	NDDKNLKTF	14.199	14.199	23	45553.7	175	160	95	249	679
Db-191	PBANKA_123350	GNEPNCINM	18.632	3.894	19	39755.9	70	187	191	232	680
Db-192	PBANKA_090210	NCYKNLMNF	18.583	1.2	21	39716.1	72	219	159	231	681
Db-193	PBANKA_083100	SGYVNFFNK	17.004	2.851	17	19922.8	115	197	207	165	684
Db-194	PBANKA_103100	KNINNYDLK	20.835	0.929	16	34996.4	35	224	211	217	687
Db-195	PBANKA_145490	KLCENDNKI	14.19	3.859	23	36546.9	176	190	103	222	691
Db-196	PBANKA_121680	NDNKNLKII	16.262	1.646	22	36244.4	127	209	137	220	693
Db-197	PBANKA_090210	KKGYNMDDF	17.647	0.178	22	37610.3	91	241	139	225	696
Db-198	PBANKA_145490	KKINNGNVV	15.97	1.354	19	15146.5	138	216	194	150	698
Db-199	PBANKA_020970	SAVDNFGNT	8.714	285.12	19	28317.8	243	78	186	192	699
Db-200	PBANKA_103100	CVLNNLIYR	15.205	34.243	15	28125.8	155	138	217	191	701
Db-201	PBANKA_031040	YMCFACLYI	13.509	13.936	14	8162.1	195	161	228	119	703
Db-202	PBANKA_090210	YAIYNCYKN	15.79	4.428	15	18112.8	141	184	219	161	705
Db-203	PBANKA_021430	CVILNIVST	12.285	342.429	13	23351.1	220	70	235	183	708
Db-204	PBANKA_093640	RHDKNEKNL	17.173	3.886	20	42096.1	108	189	175	239	711
Db-205	PBANKA_081890	FEELNDDLF	14.256	0.002	22	14101.5	173	254	140	145	712
Db-206	PBANKA_020970	CGLTNRKET	10.955	218.63	17	29052.4	226	87	205	194	712

Db-207	PBANKA_131980	KCIQNCEFK	18.9	1.689	17	40062.4	63	208	208	234	713
Db-208	PBANKA_040290	FILNNLCVQ	17.436	1.08	15	23219.9	97	222	221	181	721
Db-209	PBANKA_100320	CEIFNICTK	20.929	0.111	14	33570.2	32	246	233	213	724
Db-210	PBANKA_092610	YSPHFAHLL	13.779	13.02	17	20230.5	192	163	206	170	731
Db-211	PBANKA_100320	SHLNNRKLT	14.659	97.169	15	40582.3	164	117	216	236	733
Db-212	PBANKA_121680	NAIDNDDNY	13.826	25.084	18	30032.9	191	150	199	197	737
Db-213	PBANKA_131980	FNYEFITTL	14.336	0.924	18	12488.2	170	225	203	141	739
Db-214	PBANKA_112270	NANICSLYL	13.978	8.4	15	19957.8	183	177	218	167	745
Db-215	PBANKA_040290	HINHNFYLT	18.777	3	11	42064	66	195	247	238	746
Db-216	PBANKA_123350	WLYPNFNRF	16.919	0.244	20	32606.5	117	240	182	209	748
Db-217	PBANKA_131980	IRANNCYII	14.212	14.212	19	37984.3	174	159	189	226	748
Db-218	PBANKA_020970	LQKNNNKII	9.085	151.495	19	33572.9	239	109	187	214	749
Db-219	PBANKA_103100	CVYPNFNMK	17.897	9.512	9	42507.1	86	172	251	241	750
Db-220	PBANKA_103100	MKENNFNKL	17.593	0.278	20	42863	92	238	181	242	753
Db-221	PBANKA_100300	SCYCNYYNN	15.566	3.168	12	20260	146	194	243	171	754
Db-222	PBANKA_083100	QNINGIFYI	16.119	2.328	14	27218.5	133	202	229	190	754
Db-223	PBANKA_103100	RILNNISKF	16.73	0.443	19	32030.8	122	234	195	207	758
Db-224	PBANKA_021430	RINNLFEYI	17.028	1.394	13	31262.3	112	215	238	202	767
Db-225	PBANKA_123350	FGNYLCQKL	17.105	0.62	15	35938.9	110	231	223	219	783
Db-226	PBANKA_145490	IQQNNDFLF	14.083	1.503	20	33551.5	181	213	179	212	785
Db-227	PBANKA_083100	YSCHKYLLL	14.852	1.302	13	23078.6	160	217	239	179	795
Db-228	PBANKA_100300	NTVNNEDIK	15.499	2.007	13	34270.8	149	203	236	215	803
Db-229	PBANKA_100320	EHYYNVNNL	13.128	4.032	19	37483	211	186	190	224	811
Db-230	PBANKA_090210	DCIHNSDNY	17.455	0.307	14	45630.7	96	236	230	251	813
Db-231	PBANKA_021430	QCIINSVHA	13.103	21.6	14	36363.9	215	153	227	221	816
Db-232	PBANKA_021430	FADCFRDIF	13.504	0.018	14	11795.3	197	251	234	135	817
Db-233	PBANKA_090210	FLDNNLYTR	16.179	0.035	16	38183.8	132	249	212	227	820
Db-234	PBANKA_093640	FLPDKDKYM	15.948	0.058	18	38279.5	140	248	204	228	820
Db-235	PBANKA_021430	ACLKCFLNL	13.509	6.048	16	40962.8	196	182	210	237	825
Db-236	PBANKA_083100	CGNNNGGCD	14.031	28.512	12	46973.9	182	147	242	254	825

Db-237	PBANKA_145490	CNNNNSCEK	17.016	0.871	12	44797.1	113	226	244	247	830
Db-238	PBANKA_021430	FRDIFIDFM	14.159	0.008	15	21391	178	253	226	174	831
Db-239	PBANKA_071190	CIDYFRDTL	16.92	0.66	13	44052.8	116	230	240	246	832
Db-240	PBANKA_131980	YLNKKYFNI	13.424	0.116	11	19756.7	200	245	250	164	859
Db-241	PBANKA_093640	KKNKFFDVI	14.316	0.175	15	38949.2	171	242	224	230	867
Db-242	PBANKA_090210	AIYNCYKNL	15.376	0.84	11	46315.3	152	227	249	252	880
Db-243	PBANKA_100300	ISGNNEANK	13.121	1.641	13	37052.3	213	210	237	223	883
Db-244	PBANKA_131980	FDPNNNFLN	13.91	0.017	16	39966.1	186	252	213	233	884
Db-245	PBANKA_100300	FINPFPYSI	13.168	0.588	12	31092.1	209	232	245	201	887
Db-246	PBANKA_092610	WLGNNDDKN	14.477	0.283	13	45039.6	167	237	241	248	893
Db-247	PBANKA_093640	YVNWKEKNI	14.428	0.346	9	42441.2	169	235	252	240	896
Db-248	PBANKA_090210	MNFYNYFTS	13.836	0.475	12	38870.3	189	233	246	229	897
Db-249	PBANKA_040320	KNNNNDDSY	13.832	0.253	14	43665.5	190	239	231	244	904
Db-250	PBANKA_100320	NCDICSETI	14.179	0.164	14	46445.3	177	243	232	253	905
Db-251	PBANKA_031040	MCFACLYIL	13.08	1.21	11	44039.9	216	218	248	245	927
Db-252	PBANKA_134010	AHGNKMVLL	13.123	0.134	15	45597.8	212	244	225	250	931
Db-253	PBANKA_131980	RANNCYIIF	13.305	0.06	9	40494.3	205	247	253	235	940
Db-254	PBANKA_103100	FNLHFFFKT	13.266	0.024	5	43144	207	250	254	243	954
